# Self-Disproportionation-Induced H‑Adsorption/Desorption Zones in Amorphous Nickel Boride Cocatalyst for Efficient Photocatalytic Hydrogen Evolution

**DOI:** 10.1021/jacs.6c01844

**Published:** 2026-04-17

**Authors:** Haoyu Long, Ruina Li, Chuanbiao Bie, Jianjun Zhang, Jiaguo Yu, Hermenegildo García, Huogen Yu

**Affiliations:** † Laboratory of Solar Fuel, Faculty of Materials Science and Chemistry, 12564 China University of Geosciences, Wuhan 430078, P.R. China; ‡ Instituto Universitario de Tecnología Química, CSIC-UPV, Universitat Politècnica de València, Valencia 46022, Spain

## Abstract

Hydrogen spillover is a well-established strategy for enhancing hydrogen evolution kinetics; however, in conventional binary-component catalysts, its efficiency is often limited by long diffusion distances and significant interfacial resistance. Herein, we developed an efficient intraparticle hydrogen spillover pathway in isolated amorphous nickel boride (a-NiB) cocatalysts through an amorphization-induced self-disproportionation strategy, which significantly enhances H_2_-evolution kinetics. Combined experimental and theoretical results demonstrate that amorphization induces uneven compression and stretching of Ni–B bonds, leading to the electronic density disproportionation of nickel active sites. This effect creates spatially separated electron-deficient and electron-rich microzones within each a-NiB nanoparticle, promoting efficient hydrogen adsorption and desorption, respectively, and thereby enabling facile intraparticle hydrogen spillover within the a-NiB cocatalyst. When coupled with CdS photocatalysts, the a-NiB cocatalyst enables vigorous visible-light-driven H_2_ evolution with macroscopic bubble formation and achieves a quantum efficiency of 53%. This work redefines catalyst design in amorphous materials and opens new frontiers for energy synthesis technologies.

## Graphical abstract

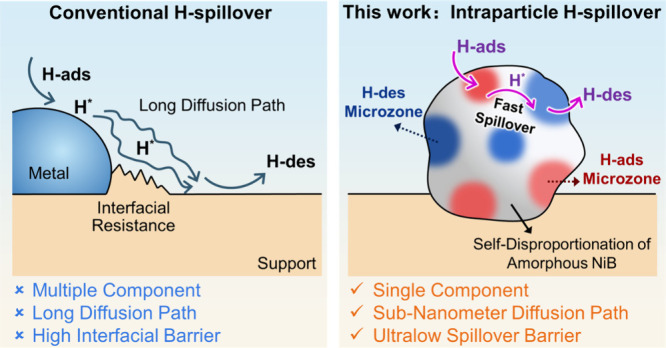

## 1 Introduction

Hydrogen (H_2_), an exceptional energy carrier offering both high energy density and zero-emission potential, necessitates accelerated development as a sustainable alternative to conventional energy systems.^1,2^ Photocatalytic conversion of solar energy into hydrogen stands as a compelling pathway to address the increasingly urgent challenges of global energy scarcity and environmental crisis.^3^ However, the solar-to-hydrogen efficiency of current photocatalytic systems remains severely constrained by the sluggish interfacial H_2_-evolution reactions, originating from the inferior H_2_-evolution kinetics of the active sites.^4,5^ Accelerated H_2_-evolution kinetics require simultaneously facilitated H-adsorption and H-desorption (H_ads_/H_des_) processes, yet conventional catalytic centers suffer from unbalanced H_ads_/H_des_ due to suboptimal electronic configurations.^6–8^ Consequently, rational electronic state engineering of active sites to realize simultaneously promoted H_ads_/H_des_ processes represents a critical pathway toward overcoming fundamental limitations in photocatalytic H_2_ evolution.^9,10^

Classical Sabatier theory targets catalyst active sites with intermediate hydrogen binding strength, characterized by near-zero hydrogen adsorption free energy (Δ*G*_H_ ≈ 0) to balance adsorption and desorption kinetics, as displayed in Figure 1a.^11,12^ However, this framework focuses exclusively on static behaviors at isolated sites, creating an intrinsic limitation where single catalytic centers cannot simultaneously achieve strong hydrogen capture and rapid hydrogen release. Recent advances reveal that efficient catalytic hydrogen evolution requires dynamic processes beyond individual sites, notably cooperative intersite interactions enabling hydrogen spillover.^13^ Such hydrogen spillover systems overcome Sabatier trade-offs by spatially segregating H-adsorption and H_2_ desorption steps across distinct catalytic sites. Current hydrogen spillover models predominantly employ binary component metal–support structures, proceeding via three concerted steps: (1) strong proton adsorption on metal sites (Δ*G*_H‑metal_ < 0), (2) interfacial H atom diffusion/spillover to adjacent supports, and (3) efficient H_2_ desorption from support sites (Δ*G*_H‑support_ > 0), as shown in Figure 1b.^14,15^ However, the H_2_-evolution kinetics in conventional hydrogen spillover systems are inherently limited by prolonged diffusion pathways (typically >5 nm) and interfacial resistances arising from Schottky barriers or lattice mismatches, which collectively elevate activation energies and suppress H_2_-evolution performance.^16^ Therefore, achieving intraparticle hydrogen spillover with subnanometer diffusion paths (<1 nm) and ultralow energy barriers (<0.3 eV) holds great potential to enhance H_2_-evolution dynamics, while such designs remain critically underexplored.

**Figure 1:**
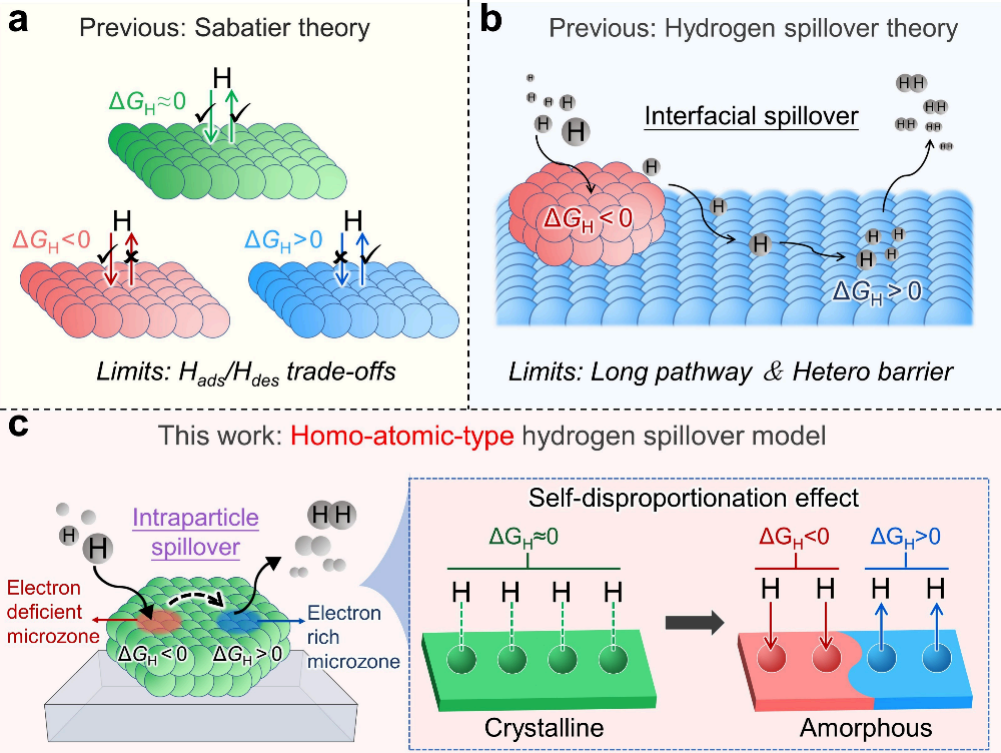
Schematic and the concept of this work. (a) Schematic diagram illustrating the Sabatier theory. (b) Illustration of the conventional hydrogen spillover models. (c) Achieving an intraparticle hydrogen spillover via amorphous-induced self-disproportionation for promoted H_2_ evolution.

Herein, we propose a groundbreaking amorphization-induced self-disproportionation strategy to achieve intraparticle hydrogen spillover in amorphous nickel boride (a-NiB). Herein, the self-disproportionation effect refers strictly to the disproportionation of the electronic density rather than the valence states. This approach creates spatially segregated subnanometer zones within individual particles with divergent hydrogen adsorption energetics (Δ*G*_H_ < 0 for adsorption zones; Δ*G*_H_ > 0 for desorption zones), establishing a first-of-its-kind homoatomic hydrogen spillover pathway that circumvents traditional bifunctional limitations (Figure 1c). Through X-ray absorption spectroscopy and first-principles calculations, we demonstrate that amorphization induces bond disproportionation (compressive/tensile Ni–B bonds) vs crystalline NiB (c-NiB), modulating Ni_*d*_-B_*p*_ orbital hybridization to generate atomic Ni sites with split *d*-band centers (−1.67 to −2.63 eV for a-NiB vs constant −2.19 eV for c-NiB). This self-disproportionation effect spatially segregates H-adsorption and desorption zones at the subnanometer scale, enabling low-barrier atomic hydrogen spillover to elevate the overall H_2_-evolution kinetics. When coupled with a semiconductor photocatalyst CdS substrate, the a-NiB cocatalyst drives vigorous visible-light-driven hydrogen production with macroscopically observable H_2_-bubble evolution, achieving a remarkable quantum efficiency of 53%. This work redefines the design principles for hydrogen evolution catalysts by achieving optimal kinetics performance in a homoatomic-type hydrogen spillover, establishing foundational frameworks for advanced catalytic energy conversion technologies.

## 2 Results and Discussion

### 2.1 Synthesis and Characterizations of CdS/a-NiB Photocatalysts

Uniform deposition of amorphous nickel boride (a-NiB) nanoparticles on CdS substrates proceeds through a light-induced autocatalytic pathway, involving photon-triggered nucleation followed by self-deposition, as revealed in Figure S1. The experimental section is detailed in the Supporting Information. Upon a first light irradiation of a homogeneous aqueous suspension containing CdS particles, nickel acetate (Ni­(CH_3_COO)_2_), and dimethylamine borane (CH_3_CH_2_NH·BH_3_, DMAB), photogenerated electrons generated from CdS can trigger the reaction of Ni^2+^ and DMAB, leading to the formation of nickel boride (NiB) nucleation sites on the CdS surface.^17,18^ Following cessation of illumination, the preformed NiB nuclei autocatalytically induce the reaction of residual Ni^2+^ and DMAB, leading to in situ growth of amorphous nickel boride (a-NiB) cocatalysts on CdS surfaces.^19^ During this process, boron atoms homogeneously incorporate into the nickel lattice, inducing Ni–B bond distortion that drives the formation of an amorphous phase, ultimately yielding CdS/a-NiB heterostructures.^20^ Based on the color evolution observed during the autocatalytic deposition process in Figure S2, bright yellow CdS gradually turns dark, evidencing the successful loading of a-NiB cocatalyst on the CdS surface.

The microstructures of the a-NiB/c-NiB-loaded CdS photocatalysts are revealed by transmission electron microscopy (TEM). It can be observed that numerous nanoparticles (identified as the a-NiB cocatalyst, ∼20 nm) are uniformly deposited on the surface of CdS host (Figure S3a). Furthermore, based on the high-resolution TEM (HRTEM) images in Figure 2a, the cocatalyst region in CdS/a-NiB shows a disordered atomic arrangement without any obvious lattice fringes, and the corresponding selected-area fast Fourier transform (FFT) pattern shows diffused halo rings without any distinguishable diffraction spots, collectively confirming the amorphous structure of a-NiB. In comparison, the CdS/c-NiB displays crystalline cocatalyst nanoparticles with discernible lattice fringes (0.204 nm for (111) planes) and sharp diffraction spots in FFT patterns, demonstrating the crystalline structure of c-NiB cocatalyst (Figures 2b and S3b). In addition, according to the energy dispersive X-ray spectrometry (EDS) mapping images, the elemental Ni and B signals are overlapped and evenly distributed with the Cd and S signals, further demonstrating the successful synthesis of the CdS/a-NiB photocatalyst (Figure 2c). In addition, based on the ICP-OES results, the atomic ratio of Ni to B in the CdS/a-NiB sample is calculated to be approximately 1.42:1. Furthermore, based on SEM, XRD, Raman, and UV–vis results, the loading of a-NiB or c-NiB cocatalysts has a negligible impact on the morphology, crystal structure, and light absorption edge of the CdS photocatalyst (Figures S4–S7).

**Figure 2:**
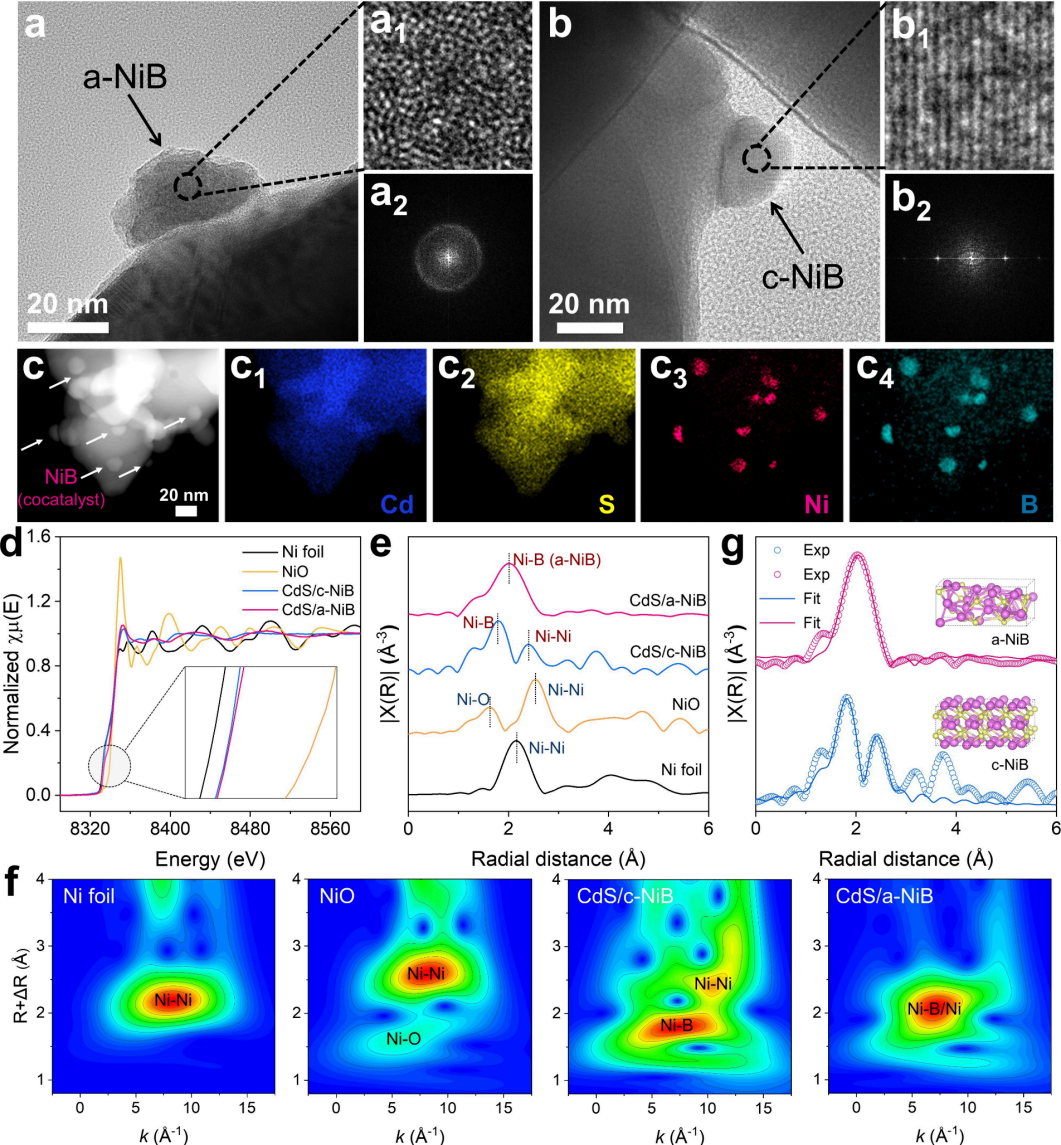
Microstructure and fine coordination characterizations. HRTEM and selected area FFT images of (a,a_1_-a_2_) CdS/a-NiB and (b,b_1_-b_2_) CdS/c-NiB. (c,c_1_-c_4_) HAADF-STEM and the corresponding EDS mapping images of CdS/a-NiB. (d) Ni k-edge XANES spectra, (e) FT-EXAFS spectra, (f) wavelet transforms of EXAFS data, and (g) fitted FT-EXAFS curves of various samples.

The electronic states and atomic-scale coordination structures of the synthesized samples are investigated using X-ray absorption fine structure spectroscopy (XAFS), as displayed in Figure 2d–g. According to the normalized Ni K-edge X-ray absorption near-edge structure spectra (XANES), the absorption edges of CdS/a-NiB and CdS/c-NiB samples are located between those of Ni foil and NiO references (with positions closer to Ni foil), suggesting the metallic Ni state in nickel boride.^21,22^ Furthermore, CdS/a-NiB and CdS/c-NiB have very close absorption edges, indicating that the amorphization has a negligible influence on the overall Ni electronic state of the nickel boride cocatalyst (Figures 2d and S8). In addition, X-ray photoelectron spectroscopy (XPS) results also show no significant shift in the Ni 2*p* and B 1*s* spectra for the CdS/a-NiB and CdS/c-NiB samples, further confirming that the overall electronic state of the nickel boride remains unchanged (Figure S9). However, despite the preservation of the overall electronic structure of the nickel boride cocatalyst after amorphization, the local electronic states of atomic Ni in the amorphous nickel boride are actually different due to the disordered coordination structure. Herein, Fourier transform extended X-ray absorption fine structure spectra (FT-EXAFS) analysis provides atomic-scale structural evidence of the ordered and disordered coordination structure in c-NiB and a-NiB cocatalysts, respectively. As shown in Figure 2e, Ni foil exhibits a dominant peak at 2.15 Å corresponding to metallic Ni–Ni bonding.^23^ Standard NiO displays two characteristic peaks at 1.62 Å (Ni–O) and 2.54 Å (Ni–O–Ni), reflecting its ordered oxide structure.^24^ Crucially, CdS/c-NiB manifests two well-defined peaks at 1.80 and 2.39 Å, assigning to crystalline Ni–B and Ni–B–Ni coordinations, respectively. Moreover, the observed distinct higher-shell signals (>3 Å) confirm the long-range crystalline order of c-NiB.^25,26^ In contrast, CdS/a-NiB exhibits only a broadened peak centered at 1.98 Å, indicating the disordered Ni–B bonding in a-NiB cocatalyst.^27^ Furthermore, its complete absence of higher-shell coordination signals unambiguously verifies the amorphous atomic configuration.^28^ The amorphous structure of a-NiB cocatalyst is further evidenced through wavelet transform EXAFS analysis (Figure 2f), where CdS/c-NiB manifests distinct intensity maxima at 7.44 Å^–1^ (Ni–B) and 10.46 Å^–1^ (Ni–B–Ni), while CdS/a-NiB shows a broadened intensity distribution centered at 6.86 Å^–1^, implying the bond-length disorder in the a-NiB cocatalyst.

Additionally, the experimental data are well-matched with the fitted results of the theoretically crystalline and amorphous nickel boride models, strongly confirming the ordered and disordered micro coordination configurations of the c-NiB and a-NiB cocatalysts, respectively (Figures 2g and S10). In summary, the above results demonstrate the successful synthesis of crystalline and amorphous NiB cocatalysts on the CdS surface to form the CdS/c-NiB and CdS/a-NiB photocatalysts, respectively.

### 2.2 Amorphization-Induced Electronic Self-Disproportionation Effect

The amorphization of nickel boride can alter the local Ni–B bond lengths, with some bonds elongating while others compress relative to the crystalline structure, as revealed in Figure 3a,b. First, the Ni–B bonds in c-NiB and a-NiB are examined using the radial distribution function (RDF). The c-NiB exhibits a sharp first-shell peak at 2.11 ± 0.01 Å, indicating a consistent Ni–B bond length of 2.11 Å in c-NiB (Figure 3a). After amorphization, the first shell Ni–B bond peak of a-NiB shows a broad range from 1.89 to 2.29 Å (2.11 ± 0.18 Å), demonstrating the prolonged and shortened Ni–B bonds in a-NiB compared to those of c-NiB (Figure 3b). Therefore, compared to the Ni–B bonds in c-NiB, the bond lengths in a-NiB exhibit a disordered distribution, with some Ni–B bonds undergoing elongation, while others experience compression (Figure S11). The local elongation or compression of the Ni–B bond length can induce an electronic self-disproportionation effect of the Ni atoms in a-NiB, as revealed by the following atom-partial density of states (DOS) results. As for c-NiB, all Ni atoms exhibit nearly the same *d*-orbital electronic configurations (Figure 3c), which is attributed to the fact that the ordered crystalline structure causes all Ni atoms to be located in the same coordination environment. However, after amorphization, the distortion of the Ni–B bonds leads to various coordination environments for each Ni atom, resulting in significantly different *d*-orbital electronic distributions in a-NiB (Figure 3d). Therefore, the total DOS of Ni 3*d* in a-NiB shows obvious degeneracy compared to the crystalline structure (Figure S12). Herein, the changes of the *d*-orbital electronic configuration across various Ni atoms can be attributed to changes in the Ni–B bond lengths in a-NiB. Based on this consideration, the Ni–B bond lengths and *d*-orbital centers of all Ni atoms in a-NiB are analyzed to investigate their relationship (Figures 3e and S13–14). All the Ni atoms in c-NiB show the same Ni–B bond length (2.11 Å) and Ni *d*-orbital center (−2.19 eV). In contrast, in a-NiB, the Ni atoms (Ni_1_ to Ni_32_ in the a-NiB model) show different Ni–B bond lengths (from 1.96 to 2.27 Å) and Ni *d*-orbital centers (from −2.63 to −1.68 eV). Therefore, an amorphization-induced self-disproportionation effect is observed: compared to the Ni atoms in c-NiB, the Ni–B bond length of Ni atoms in a-NiB undergoes a disproportionation from a constant 2.11 to a range of [2, 2.22] Å, while the *d*-orbital centers of Ni atoms also undergo a disproportionation from −2.19 eV to a range of [−2.63, −1.68] eV. Furthermore, we observe a significant linear correlation between the *d*-orbital center (*y*) and the Ni–B bond length (*x*) of Ni atoms in a-NiB (*y* = *a* + *bx*, *a* = −6.98 eV, *b* = 2.28 Å, *R* ^2^ = 0.93). Therefore, we conclude that in a-NiB, Ni atoms with weaker Ni–B bonds (longer bond lengths) exhibit higher *d*-orbital centers, while Ni atoms with stronger Ni–B bonds (shorter bond lengths) show lower *d*-orbital centers (Figure S15). Based on these two situations, compared to the Ni atoms in c-NiB (Ni_(c)_), we classify the Ni atoms in a-NiB into two categories: one with weak Ni–B bonds and high *d*-orbital centers (Ni_(a‑ads)_), and the other with strong Ni–B bonds and low *d*-orbital centers (Ni_(a‑des)_) (Figure 3f). Overall, these results demonstrate that the amorphization of nickel boride can induce local changes in the Ni–B bond length, leading to a self-disproportionation effect in the Ni atoms, ultimately forming Ni atoms with different electronic configurations in a-NiB.

**Figure 3:**
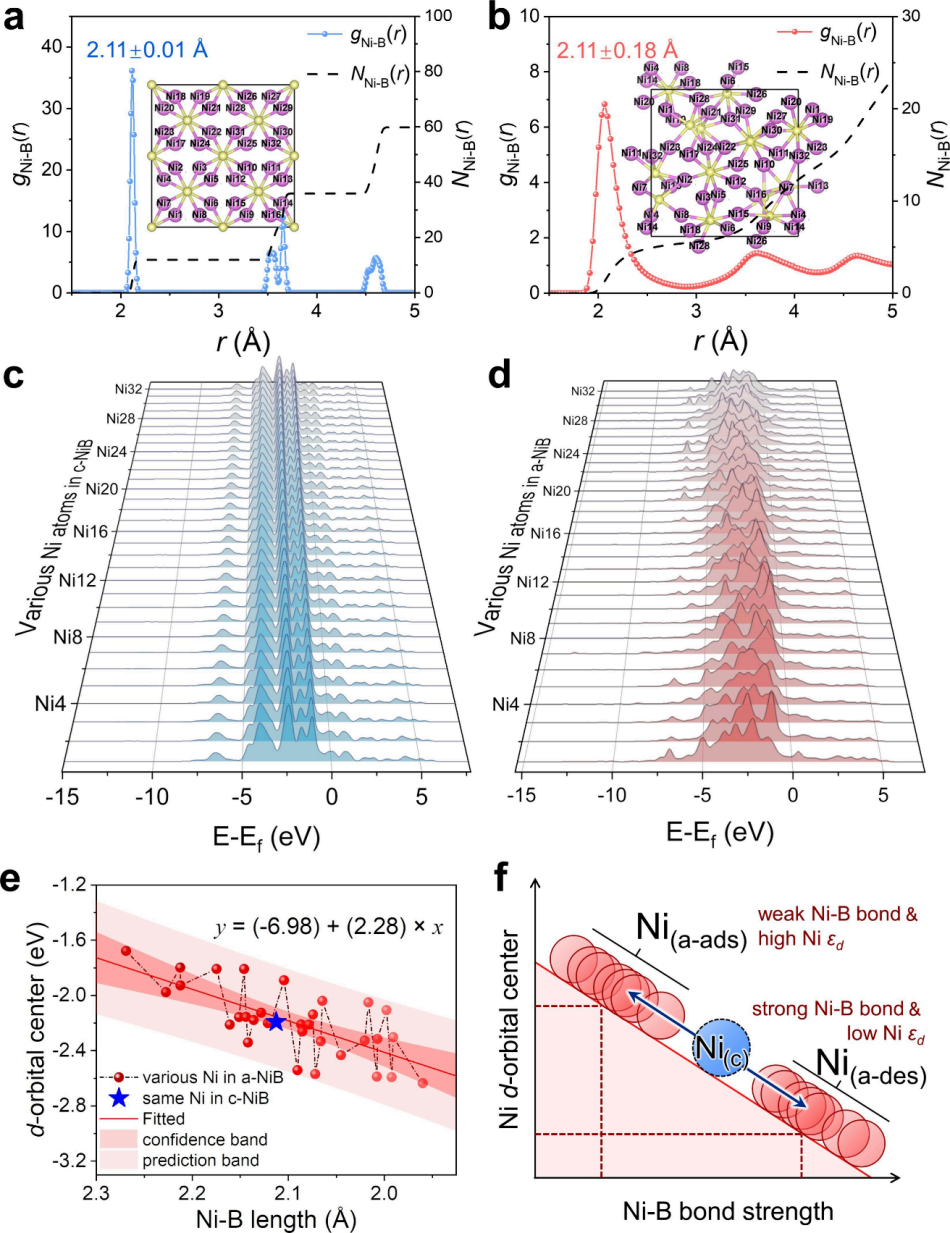
Amorphization-induced electronic self-disproportionation effect. RDF data of (a) c-NiB and (b) a-NiB. Atomic-separated DOS data for (c) c-NiB and (d) a-NiB. (e) Relationship between the Ni–B bond length and the *d*-orbital center of Ni in a-NiB. (f) Illustration for the self-disproportionation of Ni atoms in a-NiB compared to that of c-NiB.

### 2.3 Spatial-Separated H-Adsorption and H-Desorption Zones

The amorphization-induced self-disproportionation effect in the a-NiB cocatalyst can simultaneously create spatially separated electron-deficient and electron-rich zones on the a-NiB surface, as revealed in Figure 4. Owing to the aforementioned self-disproportionation effect, Ni atoms in a-NiB exhibit distinct *d*-orbital configurations: some possess higher *d*-orbital centers, while others show lower centers. Consequently, when H atoms adsorb onto different Ni sites on a-NiB surface, the formed antibonding orbital occupancy varies significantly: at Ni sites with high *d*-orbital centers (Ni_(a‑ads)_), the decreased antibonding orbital occupancy strengthens Ni–H_ad_ bonding, while at Ni sites with low *d*-orbital centers (Ni_(a‑des)_), the increased antibonding occupancy weakens H-adsorption (Figure 4a,b).^29,30^ Therefore, the heterogeneous adsorption strengths at different Ni sites create a dual-functional a-NiB surface: specific regions facilitate H-adsorption, while others promote H-desorption. This behavior is further investigated through the following electronic localization function (ELF) simulation and site-specific hydrogen-adsorption free energy (Δ*G*_H_) calculations. According to the ELF data in Figure 4c, the c-NiB surface shows a periodic electron density distribution with each Ni atom exhibiting the same electron density. After amorphization, the electron density distribution on the a-NiB surface becomes disordered, with some Ni atoms showing increased electron density and others showing decreased electron density (Figure 4d). As a result, spatially separated electron-deficient (orange) and electron-rich (cyan) regions can be clearly found on the a-NiB surface (Figure 4e).

**Figure 4:**
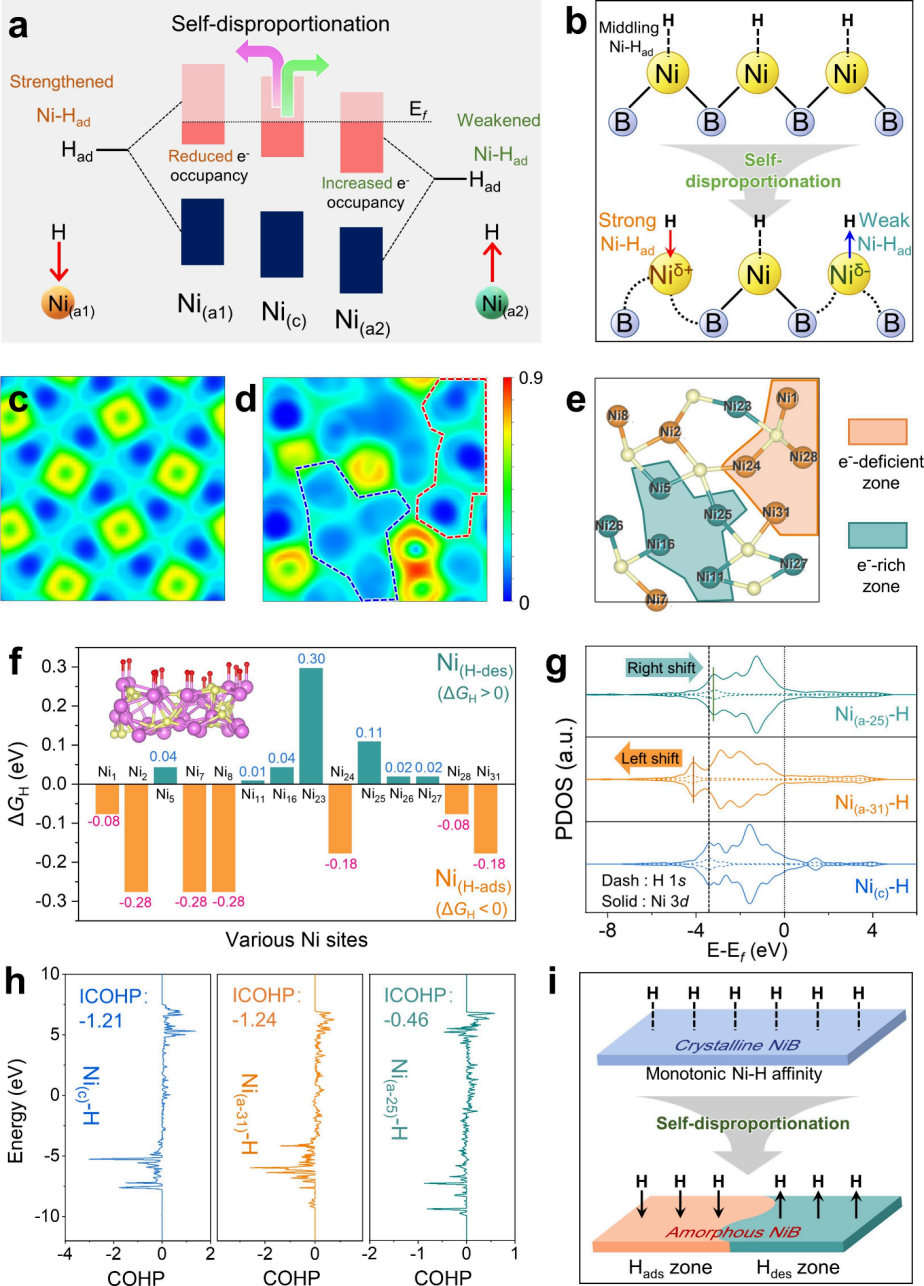
Spatially separated H-adsorption and H-desorption zones. Diagram illustrating the self-disproportionation effect of (a) antibonding-orbital occupancy, (b) Ni electronic states, and Ni–H_ad_ bond strength. Surface planar ELF data of (c) c-NiB and (d) a-NiB. (e) Corresponding atomic arrangement of the a-NiB surface. (f) Calculated Δ*G*_H_ values of various Ni atoms on the a-NiB surface. (g) PDOS and (h) COHP data of the Ni_(c)_-H, Ni_(31)_-H, and Ni_(25)_-H. (i) Schematic diagram for the formation of H_ads_ and H_des_ zones in amorphous NiB via a self-disproportionation effect.

The electron-deficient and electron-rich regions on the a-NiB surface serve as H-adsorption and H-desorption zones during the H_2_-evolution process, respectively. Herein, the H-adsorption abilities of the electron-deficient and electron-rich regions are further revealed via calculating the Δ*G*_H_ of various Ni atoms on the a-NiB surface, and the results are shown in Figure 4f. It is obvious that at electron-deficient regions of a-NiB surface, Ni atoms exhibit Δ*G*_H_ < 0 (−0.18 to −0.08 eV), demonstrating thermodynamically favorable H-adsorption. Conversely, Ni atoms in electron-rich regions show Δ*G*_H_ > 0 (0.01–0.11 eV), indicating their favorable H-desorption ability.^31^ For comparison, c-NiB displays a uniform hydrogen affinity with a constant Δ*G*_H_ value of −0.12 eV across all sites, indicating a homogeneous strength of H-adsorption. Therefore, the spatially separated H-adsorption (H_ads_) and H-desorption (H_des_) zones are synchronously constructed on the a-NiB surface. Therefore, unlike the uniform active sites on c-NiB, the a-NiB surface undergoes an amorphization-induced electronic self-disproportionation. This effect inherently shifts the local *d*-band centers, simultaneously creating H-ads zones with stronger hydrogen binding (lower Δ*G*_H_) and H-des zones with weaker hydrogen binding (higher Δ*G*_H_) (see details in Figure S16 of the Supporting Information). In addition, the strong H-adsorption of H_ads_ zones and weak H-adsorption of H_des_ zones are further demonstrated by the following projected density of state (PDOS) and crystal orbital Hamiltonian population (COHP) analyses.^32,33^ With H-adsorption on c-NiB, an obvious Ni_3*d*_-H_1*s*_ hybridization peak emerges at −3.41 eV, confirming successful Ni–H bonding on the c-NiB surface (Figures 4g and S17). Upon amorphization, Ni sites of H_ads_-zones exhibit a negative shift of this hybridization peak (−3.41 to −4.14 eV), indicating enhanced Ni–H stability and stronger H-adsorption. Conversely, H_des_-zone sites display a positive peak shift (−3.41 to −3.18 eV), showing Ni–H destabilization and weak H-adsorption. The above findings align precisely with COHP calculations results: H_ads_-zone Ni sites exhibit lower ICOHP values than c-NiB (−1.24 vs −1.21), while H_des_-zone Ni sites show significantly higher ICOHP (−0.46 vs −1.21) (Figure 4h). This confirms that hydrogen adsorption is energetically favored at the H_ads_ zones and readily desorbs from the H_des_ zones. These results demonstrate that hydrogen atoms are more easily adsorbed on the H_ads_ zones, whereas the H_des_ zones facilitate hydrogen desorption in the a-NiB cocatalyst. Consequently, the self-disproportionation in a-NiB yields spatially separated H_ads_ and H_des_ zones, endowed with enhanced H-adsorption efficiency and facilitated H-desorption capability, respectively (Figure 4i).

### 2.4 Intraparticle Hydrogen Spillover in a-NiB

During the hydrogen evolution process, the simultaneous existence of H-adsorption and H-desorption zones on a-NiB is experimentally evidenced by in situ attenuated total reflection surface-enhanced infrared absorption spectroscopy (ATR-SEIRAS). Comparative analyses of in situ ATR-SEIRAS spectra for c-NiB and a-NiB are displayed in Figure 5a,b. Both c-NiB and a-NiB show distinct signals at 3900–3000 cm^–1^, corresponding to the water-related features.^34–36^ In addition, the Ni–H stretching vibrations are at 2020 cm^–1^, and the Ni–OH stretching vibrations are at 1865 cm^–1^. Critically, the Ni–H stretching band of amorphous a-NiB exhibits significant broadening (fwhm = 85 cm^–1^), which is substantially wider than the single sharp peak observed for crystalline c-NiB (fwhm = 32 cm^–1^). This peak broadening emerges from the coexistence of strong and weak adsorption sites induced by electronic self-disproportionation: spectral overlap between strongly adsorbed hydrogen on electron-deficient Ni^δ+^ sites (across between 2060 and 2020 cm^–1^) and weakly adsorbed hydrogen on electron-rich Ni^δ‑^ sites (across between 2020 and 1960 cm^–1^), in precise agreement with theoretical predictions (Figure 5c). Consequently, we experimentally confirm the coexistence of strong H-adsorption zones (H_ads_ zones) and weak H-adsorption zones (H_des_ zones) within amorphous a-NiB during the H_2_-evolution process.

**Figure 5:**
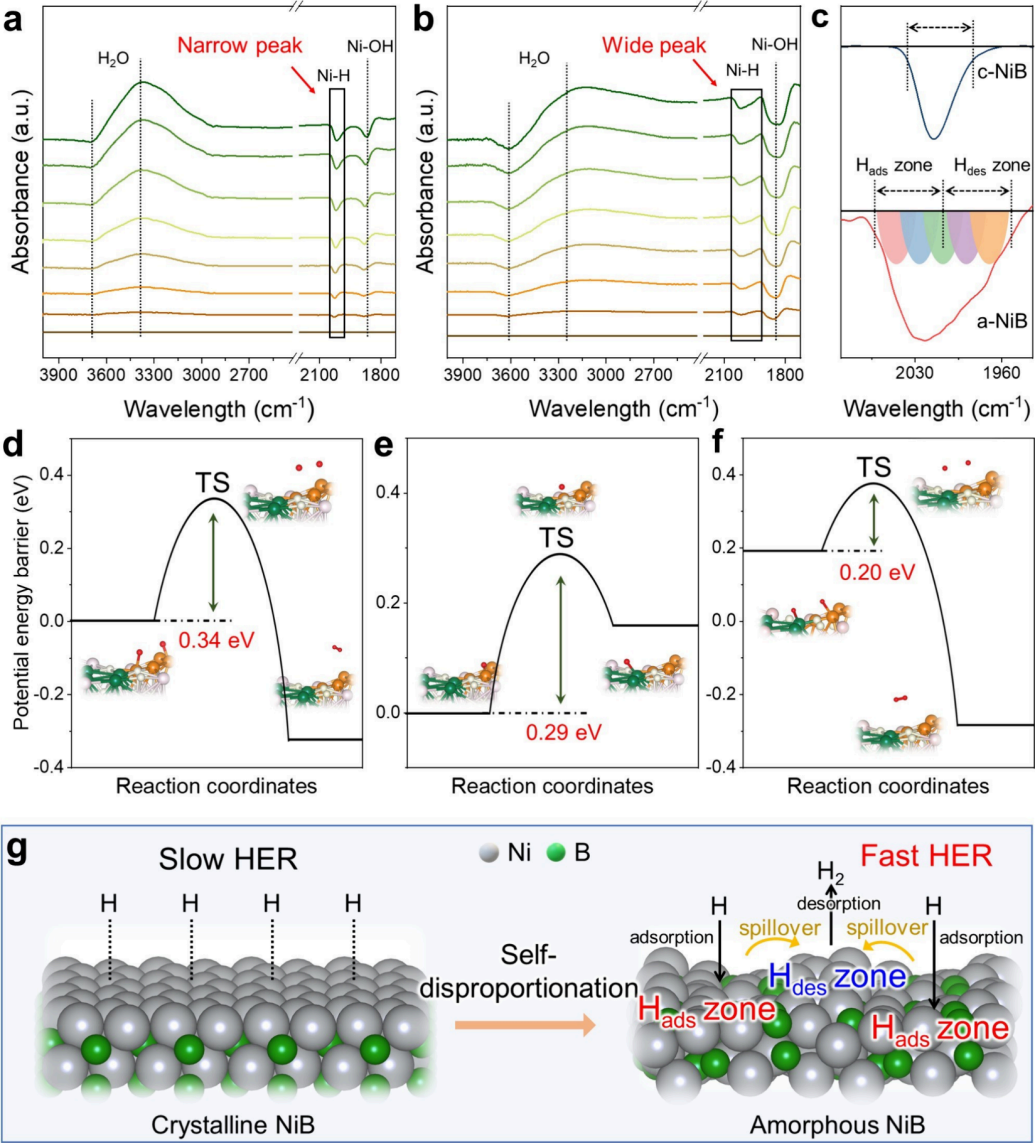
Intraparticle hydrogen spillover over the H_ads_ and H_des_ zones in a-NiB. In situ ATR-SEIRAS of (a) c-NiB and (b) a-NiB. (c) Schematic illustration of peak broadening caused by the coexistence of strong and weak H-adsorption sites. Kinetic energy barrier profile of (d) H-desorption at H_ads_ zone, (e) H spillover from H_ads_ zone to H_des_ zone, (f) H-spillover and H-desorption process. (g) Illustration for promoted H_2_ production via intraparticle hydrogen spillover in a-NiB cocatalyst.

The spatially separated H_ads_ and H_des_ zones in a-NiB enable directional intraparticle hydrogen spillover from H_ads_ zones to H_des_ zones, thereby accelerating H_2_-evolution kinetics in photocatalytic processes. To validate the feasibility of this spillover pathway, the associated reaction barriers of the H-spillover processes are rigorously calculated using climbing-image nudged elastic band (CINEB) methodology (Figure 5d–f).^37–39^ It can be observed that the hydrogen spillover from the H_ads_ zone to the H_des_ zone exhibits a low energy barrier of 0.29 eV (Figure 5e), which is obviously lower than the energy barrier for direct hydrogen desorption on the H_des_ zone (0.34 eV) (Figure 5d). Such results indicate that the hydrogen accumulation on H_ads_ zones is more tend to undergo kinetic transfer to H_des_ zones rather than desorbing directly from the H_ads_ zones. Furthermore, after hydrogen spillover from the H_ads_ zone to the H_des_ zone, the energy barrier for subsequent H_2_ desorption on the H_des_ zone is significantly reduced to 0.20 eV (Figure 5f). Thus, these findings strongly prove that the hydrogen transfer from H_ads_ zones to H_des_ zones and its subsequent desorption on H_des_ zones are more favorable for the H_2_-evolution process on the a-NiB surface. According to the above results, a synergistic homoatomic-type hydrogen spillover model is suggested to enhance H_2_-evolution kinetics of a-NiB cocatalyst (Figure S18). Specifically, in the H_2_-evolution process, the protons in solution can be effectively captured and accumulated on H_ads_ zones via the Ni–H_ad_ interaction. Subsequently, the adsorbed H atoms on the H_ads_ zones can be easily migrated to the H_ads_ zones via a kinetically advantageous process. Ultimately, the transferred hydrogen atoms on the H_des_ zones combine into H_2_, which is easily desorbed to produce free H_2_ molecules. Therefore, the above procedures can realize a homoatomic-type H spillover process, simultaneously realizing fast hydrogen adsorption and easy hydrogen desorption, thereby boosting the overall H_2_-evolution kinetics of a-NiB (Figure 5g).

### 2.5 Promoted Photocatalytic H_2_-Evolution Performances

The photocatalytic H_2_-production activity of various photocatalysts is accessed under 420 nm LED light irradiation, and the results are displayed in Figure 6. Bare CdS shows a low H_2_-production rate of 0.45 mmol g^–1^ h^–1^, due to serious photoelectron–hole recombination and lack of active sites. With the loading of amorphous NiB cocatalyst on CdS surface, the CdS/a-NiB photocatalysts with various a-NiB loading amounts (1, 3, 5, 8, 10, and 12 wt %) possess remarkably enhanced H_2_-evolution rates and show a volcano-plot trend (Figure 6a). Specifically, the optimum CdS/a-NiB (with 8 wt % a-NiB loading amount) owns an ultrahigh photocatalytic H_2_-generation rate of 12.81 mmol g^–1^ h^–1^, exhibits an apparent quantum yield (AQY) of 53% (TON ≈ 201), which surpasses other state-of-the-art cocatalyst-modified CdS photocatalysts (Table S1). Importantly, under simulated sunlight irradiation (white light), H_2_ bubbles are continuously observed emerging on the surface of CdS/a-NiB samples (Figure 6b), further confirming the high photocatalytic H_2_-production efficiency of the a-NiB cocatalyst. In addition, cycling performance tests confirm that CdS/a-NiB maintains high photocatalytic hydrogen evolution activity and remains a stable structure after testing (Figures 6c and S19). It is worth noting that the H_2_-production performance of CdS/a-NiB is apparently higher than that of CdS/c-NiB (7.51 mmol g^–1^ h^–1^), proving the boosted H_2_-evolution kinetics through the amorphization-induced self-disproportionation strategy in a-NiB cocatalyst. Furthermore, the amorphization of the NiB cocatalyst enhances the H_2_-evolution activity of TiO_2_ and g-C_3_N_4_ (Figure 6d), demonstrating the broad applicability of amorphous NiB.

**Figure 6:**
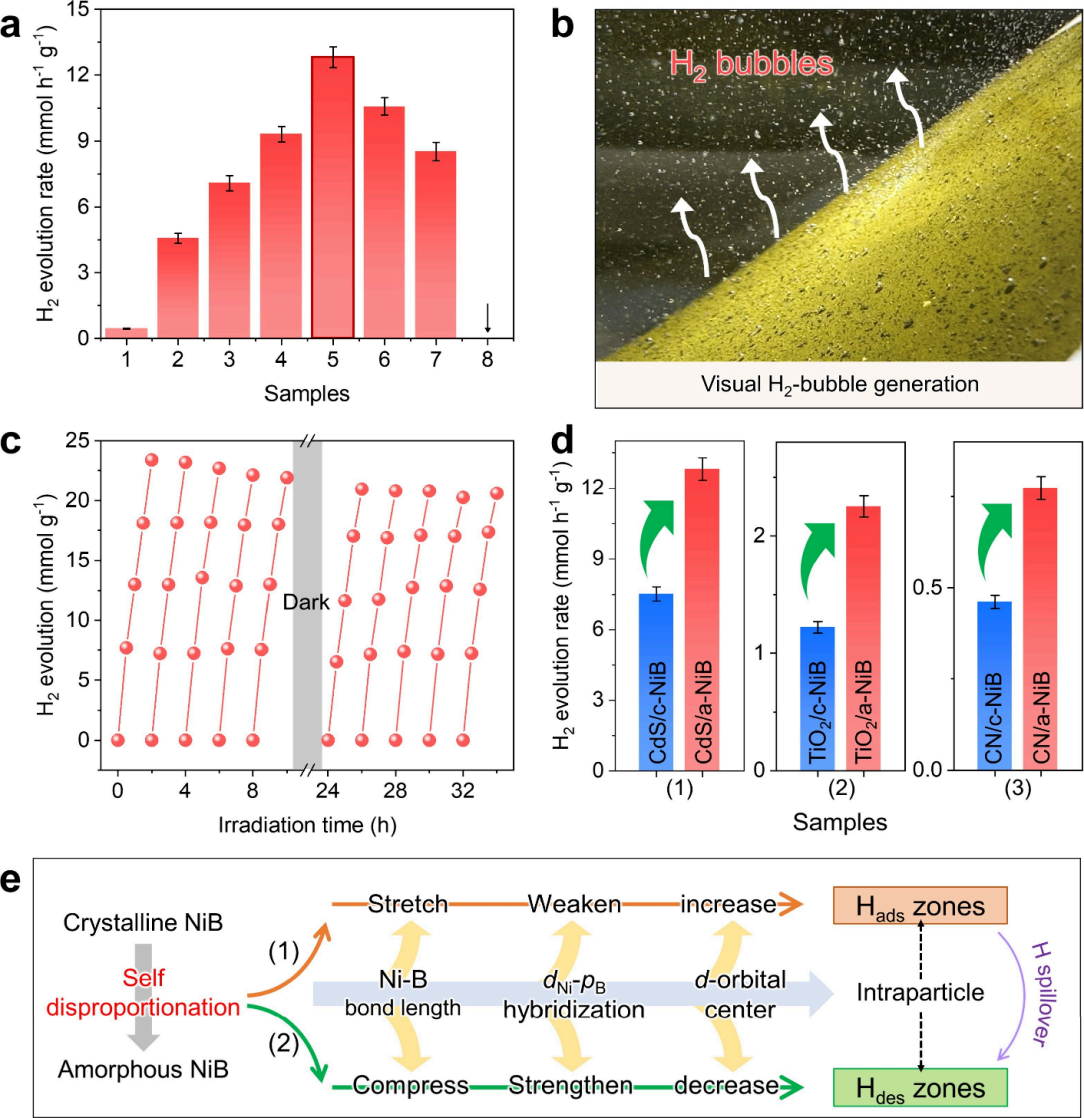
Photocatalytic hydrogen evolution performance. (a) H_2_-evolution rates of (1) CdS, (2–7) CdS/a-NiB with different a-NiB amounts: (2) 1 wt %, (3) 3 wt %, (4) 5 wt %, (5) 8 wt %, (6) 10 wt %, (7) 12 wt %, and (8) pure a-NiB. (b) Photograph for the visual H_2_ bubble generation of the CdS/a-NiB photocatalyst under visible light. (c) Cycling photocatalytic H_2_-evolution tests of 10 runs for the CdS/a-NiB. (d) H_2_-evolution rates of various samples. (e) Mechanism for the amorphization-induced self-disproportionation effect to achieve intraparticle H spillover over the H-ads/des zones.

Herein, an amorphization-induced self-disproportionation mechanism is established: The amorphization induces bond disproportionation through compressive/tensile Ni–B bonds, splitting the Ni *d*-band centers into a range of −1.67 to −2.63 eV. This electronic self-disproportionation effect creates distinct H-adsorption/desorption zones within a single a-NiB nanoparticle, enabling efficient intraparticle hydrogen spillover across these H-ads/des zones (Figure 6e). As a result, this unique homoatomic-type hydrogen spillover boosts the interfacial H_2_-evolution kinetics of a-NiB cocatalyst, thereby enhancing the photocatalytic H_2_-production activity of the CdS/a-NiB.

### 2.6 Charge Carrier Transfer Dynamics

The promoted H_2_-evolution kinetics of a-NiB can also improve the spatiotemporal charge transfer dynamics of CdS/a-NiB, as revealed by in situ Kelvin probe force microscopy (KPFM) and femtosecond transient-absorption spectroscopy (fs-TAS) in Figure 7. According to the KPFM results (Figures 7a and S20–S22), the contact potential difference (CPD) values of CdS/c-NiB increase upon light irradiation, showing an average surface photovoltage (SPV) value of 199 mV (Figure 7a).^40,41^ After amorphization, CdS/a-NiB shows an increased SPV value of 452 mV (Figure 7b), which is 2.27 times higher than that of CdS/c-NiB, demonstrating the promoted electron–hole separation (Figure S23). The fs-TAS is further conducted to investigate the temporal evolution of the charge carrier in CdS/c-NiB and CdS/a-NiB. Clearly, both the CdS/c-NiB and CdS/a-NiB exhibit positive signals at 500 nm, corresponding to excited state absorption (ESA), and the negative signal observed in the 520–690 nm range is attributed to stimulated emission (SE).^42,43^ Interestingly, compared to CdS/c-NiB, CdS/a-NiB exhibits significantly reduced ESA and SE signal intensities (Figure 7c–f). This is because the rapid H_2_-evolution kinetics of a-NiB consume the photogenerated electrons and decrease the electron population in the conduction band, thereby suppressing the ESA and SE processes (Figure S24).^44,45^ Furthermore, according to the fitted decay curves, CdS/a-NiB shows a longer average electron lifetime (117.7 ps) compared to that of CdS/c-NiB (42.7 ps), suggesting more efficient electron transfer from CdS to a-NiB (Figure 7g–i). In addition, according to the photoelectrochemical measurements (transient photocurrent response, electrochemical impedance, and TRPL results in Figure S25), the CdS/a-NiB shows a maximal photocurrent density, minimum impedance, and longest fluorescent lifetime in contrast with other samples, implying the efficient separation and transportation of charges in CdS/a-NiB. Thus, the a-NiB cocatalyst not only rapidly consumes photogenerated electrons but also facilitates their transfer from CdS, ultimately leading to highly efficient photocatalytic hydrogen production.

**Figure 7:**
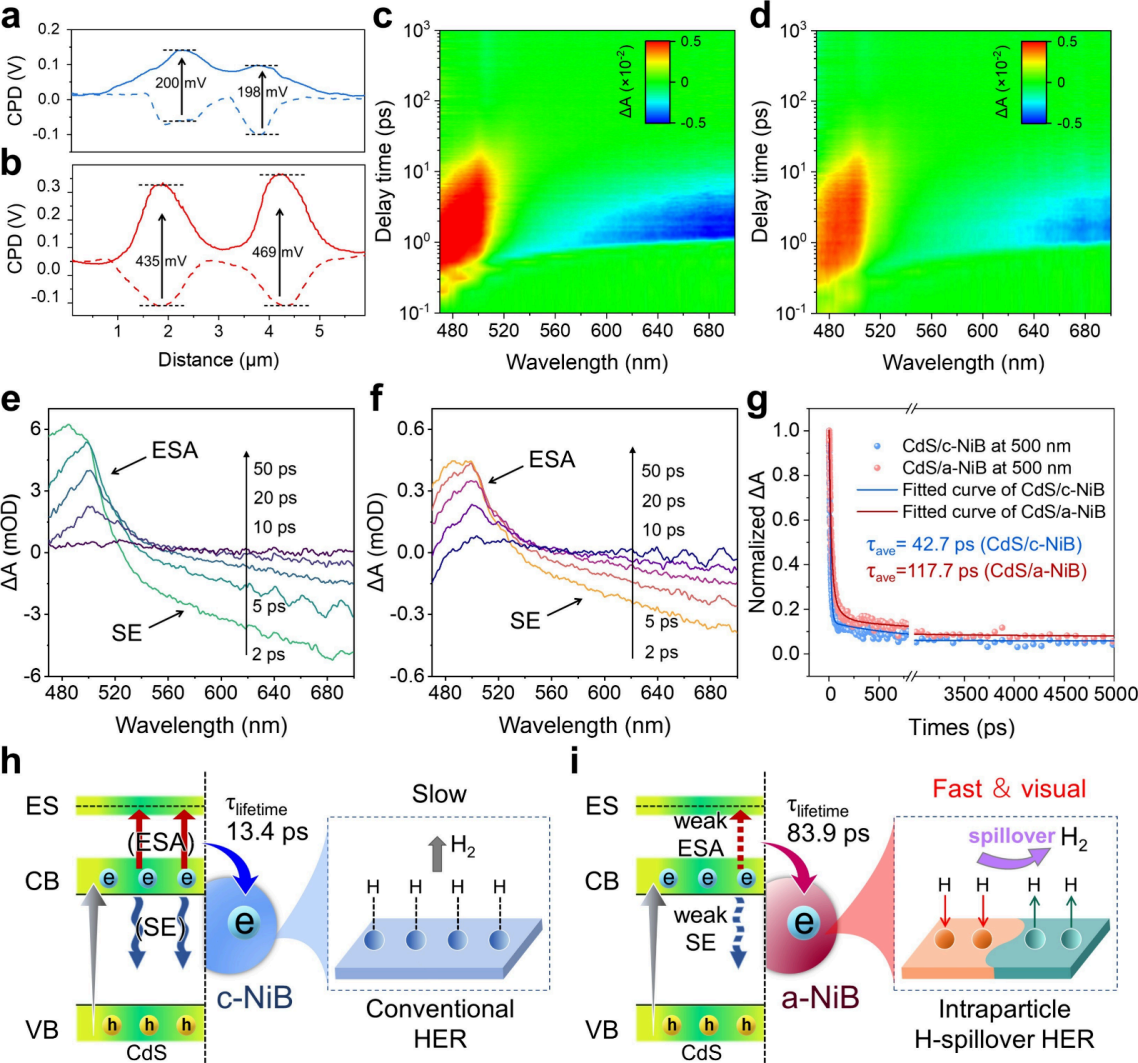
Insight into spatiotemporal charge carrier transfer dynamics. CPD curves of (a) CdS/c-NiB and (b) CdS/a-NiB under dark (dash) and irradiation (solid) conditions. Pseudocolor plots and transient-absorption signals within 50 ps of (c,e) CdS/c-NiB and (d,f) CdS/a-NiB. (g) Time-dependent decay curves of the ESA signal of the samples. (h,i) Schematic illustration of the boost charge transfer dynamics via the amorphous strategy.

## 3 Conclusions

This study demonstrates that the amorphization of nickel boride induces an electronic self-disproportionation effect, overcoming the Sabatier trade-off via intraparticle hydrogen spillover at subnanometer scales. Within individual a-NiB particles, compressive/tensile bond strains split the Ni *d*-band centers into a wide-range configuration (from −1.67 to −2.63 eV), creating spatially separated H-adsorption (Δ*G*_H_ < 0) and H-desorption (Δ*G*_H_ > 0) zones on the a-NiB surface. This atomic-scale segregation of H-ads/H-des zones enables low-barrier hydrogen spillover, significantly enhancing the H_2_-evolution kinetics. When coupled with CdS photocatalysts, the a-NiB cocatalyst achieves visible-light-driven hydrogen production with observable bubble evolution and 53% quantum efficiency, outperforming the c-NiB cocatalyst by 1.7 times. This work establishes a new material design criterion, where electronic self-disproportionation in homogeneous catalysts overcomes fundamental kinetic limitations, paving the way for transformative advancements in energy technologies.

## Supplementary Material



## Notes

Detailed experimental and characterization methods, DFT calculation parameters, photocatalytic testing protocols, and supplementary data, including SEM/TEM images, XRD patterns, Raman and XPS spectra, UV–vis absorption profiles, KPFM mappings, TRPL decay curves, and photoelectrochemical results (PDF)
