# Supplementary material for: Self-Disproportionation-Induced H‑Adsorption/Desorption Zones in Amorphous Nickel Boride Cocatalyst for Efficient Photocatalytic Hydrogen Evolution
Source: J Am Chem Soc. 2026 Apr 17;148(16):17146–56. doi: 10.1021/jacs.6c01844 (PMC13133767; doi:10.1021/jacs.6c01844)
Supplement: Supplementary file 1 [file ja6c01844_si_001.pdf]

Supporting Information

Self-Disproportionation-Induced H-Adsorption/Desorption Zones in Amorphous Nickel Boride Cocatalyst for Efficient Photocatalytic Hydrogen Evolution

Haoyu Long[1], Ruina Li[1] Chuanbiao Bie[1,*], Jianjun Zhang[1], Jiaguo Yu[1,*], Hermenegildo García[2,*] and Huogen Yu[1,*]

[1] Laboratory of Solar Fuel, Faculty of Materials Science and Chemistry, China University of Geosciences, Wuhan, 430078, P.R. China.

[2] Instituto Universitario de Tecnología Química, CSIC-UPV, Universitat Politècnica de València, Valencia 46022, Spain.

*Corresponding Author: biechuanbiao@cug.edu.cn (C. B.); yujiaguo93@cug.edu.cn (J. Y.); hgarcia@itq.upv.es (H. G.); yuhuogen@cug.edu.cn (H. Y.)

S1 Materials and Methods

CdS (99%) and DMAB ($C_2H_{10}BN$, 99%) were purchased from Aladdin Biochemical Technology Co., Ltd. (China). Nickel acetate tetrahydrate ($NiC_4H_6O_4{\cdot}4H_2O$, 99%) were obtained from Macklin Biochemical Technology Co., Ltd. (China). Triethanolamine (TEOA) and lactic acid (LA) were purchased from National Medicines Corporation Ltd. of China (China). The chemicals in this work are of analytical grade, and they are utilized without any purification.

The CdS/a-NiB is prepared by a photo-triggered auto-deposition route. First, commercial CdS (100 mg) nanoparticles were suspended in 80 mL of triethanolamine-water solution (10 vol.%) in a flask. To this, 688 μL of a mixed aqueous solution containing $Ni(CH_3COO)_2$ (0.1 mol L^{-1}) and DMAB (0.5 mol L^{-1}) was added, ensuring a Ni to CdS mass ratio of 8 wt.%. After continuous magnetic stirring, the above system was sealed with a rubber septum and degassed by purging with a N_2 atmosphere. In a photo-triggered nucleation process, the flask was exposed to 3 minutes of irradiation using four LED illuminants with a monochromatic wavelength of 420 nm, enabling the formation of a-NiB nucleation. After turning off the light, the above system is continuously stirred for 2 h under dark conditions and 25 °C to conduct the *in situ* auto-growth of a-NiB nanoparticles on CdS surface. Subsequently, the products are centrifuged, washed, and dried to gain the CdS/a-NiB sample. By changing the amount of $Ni(CH_3COO)_2$ and DMAB mix solution, various a-NiB-loading amounts of CdS/a-NiB samples can be easily obtained. In addition, the crystalline NiB-loaded CdS (CdS/c-NiB) sample is gained via adjusting the reaction temperature (80 °C) during the

self-deposition process and calcining the obtained solid in an Ar atmosphere (350℃, 2h). All the above photocatalysts could be obtained with a yield of over 95%.

S2 Fundamental Characterizations

The crystal phases of the samples were analyzed using a Shimadzu XRD-6100 X-ray diffractometer. Surface morphologies and microstructures were characterized by field emission scanning electron microscopy (FESEM, JEOL JMS-7500) and transmission electron microscopy (TEM, FEI Titan G2), respectively. Ultraviolet-visible diffuse reflectance spectra (UV-vis DRS) were recorded on a Shimadzu UV-2600 spectrophotometer. Elemental compositions were determined via ICP-OES. X-ray photoelectron spectroscopy (XPS) measurements were performed on a Thermo Fisher ESCALAB 250Xi spectrometer equipped with an Al Kα source (1486.6 eV), with all binding energies calibrated to the adventitious C 1s peak at 284.8 eV. Photo-irradiated Kelvin probe force microscopy (KPFM) was conducted using a Shimadzu SPM-9700. Time-resolved photoluminescence (TRPL) spectra were acquired on an Edinburgh FLS1000 fluorescence lifetime spectrophotometer. In situ attenuated total reflectance Fourier transform infrared (ATR-FTIR) spectroscopy was carried out using a Nicolet iS50 FT-IR spectrometer (Thermo Scientific) by Shanghai Yuanfang Technology Co., Ltd.

S3 X-ray Absorption Fine Spectroscopy

The X-ray absorption fine structure (XAFS) spectra of Ni K-edge were collected

in transmission mode on a commercial Laboratory-Based XAFS spectrometer (Table XAFS-500A, Specreation Instruments Co., Ltd.). An X-ray tube was used to generate X-ray, and the voltage and current were set to 25 kV and 20 mA, respectively. The Si (551) spherically bent crystal analyzers with a radius of curvature of 500 mm and the R250 mm Rowland circle were used to provide monochromatized X-ray beam. The data underwent intensity normalization, baseline subtraction, and Fourier transformation using the Athena software. Then the k^2-weighted R-space data were obtained. The amplitude reduction factor (S_0^2), coordination numbers (N), distances to the scattering atoms (R), and mean-squared displacements (σ^2) can be determined by fitting the R-space data using the Artemis program.

S4 Femtosecond Transient Absorption Spectroscopy Measurements

Femtosecond transient absorption spectroscopy (fs-TA) spectra were performed on a pump-probe system (Helios Fire, Ultrafast System) with a maximum time delay of 8 ns, controlled by a motorized optical delay line. An-800 nm ultrashort laser pulse was generated by an ultrafast optical parametric amplifier (Opera Solo) which was excited by a regenerative amplifier (Coherent Astrella, 800 nm, 35 fs, 7 mJ, 1 kHz), seeded with a mode-locked Ti: sapphire oscillator (Coherent Vitara-S, 800 nm, 80 MHz) and pumped with an LBO laser (Coherent Evolution-50C, 1 kHz system). The main beam was converted to a 400 nm pump pulse by an optical parametric amplifier (Opera Solo) and a neutral-density filters was used to control the average pump power at ~120 μW. The other beam was transformed into a white-light continuum (420-800 nm)

through a sapphire, which become a probe pulse. The kinetic curves of samples were fitted by the multi-exponential decay function. Samples were dispersed in pure acetonitrile (or a solution of the reaction substrates) at a concentration of 0.2 g L^{-1}. The mixtures were added into quartz cuvettes with a path length of 2 mm, and then the cuvettes were sealed using rubber septa caps and degassed with Ar for 10 min.

The decay curves obtained from the fs-TA spectra were fitted by the following multi-exponential equation:

$$I_{(t)} = \mathrm{I}_{(0)} + \sum_{i=1}^{n} A_i \exp\left(-t/\tau_i\right)$$

where $I_{(t)}$ and $I_{(0)}$ represent the transient signal intensity and baseline correction value, and t is the probe time delay. A_i and τ_i are amplitudes and decay times, respectively. The minimum number of components n to satisfactorily fit the experimental data is two.

S5 Photocatalytic Hydrogen Evolution Tests

Photocatalytic H_2-generation performance was evaluated using a three-neck Pyrex reactor (100 mL) under ambient conditions. In each test, 50 mg photocatalyst was dispersed in 80 mL aqueous solution containing 10 vol% LA as sacrificial agent. After purging with high-purity N_2 for 15 min to establish anaerobic conditions, reactions were initiated under magnetic stirring using four 420 nm LED light sources (3 W each). Evolved gases were quantitatively analyzed by sampling 0.4 mL aliquots at 30-min intervals over 2 hours via gas chromatography (Shimadzu GC-2014C, N_2 carrier gas), enabling determination of H_2-production rates.

S6 Cycling H_2-Evolution Tests

To evaluate the stability and reusability of the synthesized photocatalysts, 0.05 g of CdS/a-NiB sample was used for a cycling test to evaluate the photocatalytic H_2 generation activity in 10 consecutive runs. The first test was run and recorded for 2 hours of irradiation. After that, the suspension was re-degassed with pure N_2 for 10 min to drive away the produced H_2 in the system, and then applied for the next photocatalytic H_2-generation test.

S7 Visible H_2-Evolution Tests

The visual H_2-evolution experiment was performed with the optimal CdS/a-NiB photocatalyst. In detail, the freshly prepared CdS/a-NiB photocatalyst sample (50 mg) was directly filtered and collected on the filter paper. Subsequently, the resultant filter paper was attached to a glass substrate and put in a beaker (100 mL) with 10 vol. % lactic acid aqueous solution. Finally, the above reaction device was irradiated with a Xe lamp (300 W) for visual H_2-evolution experiment.

S8 Photoelectrochemical Measurements

Photoelectrochemical (PEC) curves were measured on an electrochemical analyzer (CHI660E, China) in a standard three-electrode configuration. The prepared samples were loaded on fluorine-doped tin oxide (FTO) conductor glass, a standard Ag/AgCl electrode and the platinum foil as the working electrodes, reference electrode and counter electrode, respectively, with Na_2SO_4 (0.5 mol L^{-1}) as the electrolyte

solution. The method of working electrodes was the same as in the previous works. Linear sweep voltammetry (LSV) curves were obtained in the potential ranging of -1.0 to -1.6 V with a scan rate of 10 mV s^{-1}. Transient photocurrent responses with time (*i-t* curves) were recorded at 0.5 V bias potential during periodic ON/OFF illumination cycles under a 3 W LED lamp (365 nm), and electrochemical impedance spectroscopy (EIS) curves were conducted at the frequency range of 0.01-10^5 Hz with an ac amplitude of 10 mV under the open-circuit voltage.

S9 Apparent Quantum Yield (AQY) Calculations

The apparent quantum yield (AQY) was calculated via the following equation:

$$\text{AQY}(\%)=\frac{\text{number of reacted eletrons}}{\text{number of incident photons}}\times 100\%$$

$$=\frac{\text{number of evolved } H_2 \text{ molecules}\times 2}{\text{number of incident photons}}\times 100\%$$

$$=\frac{2R_{H_2}t_1N_A}{EAt_2\lambda/hc}\times 100\%=6.6482\times 10^{-5}\times R_{H_2}/(E\times A\times\lambda)\times 100\%$$

R_{H_2}(mol h^{-1} g^{-1}) represents the H_2 evolution rate, E (W cm^{-2}) represents the monochromatic light intensity, A (cm^2) represents the area of light radiation for the reaction system, λ (m) represents the monochromatic wavelength, while t_1 (h), t_2 (s), h (W), and c (m s^{-1}) are constants with values of 1, 3600, 6.626×10^{-34}, and 3×10^8, respectively.

In this work, 50 mg of the photocatalyst was irradiated by four 420 nm LED lights as the light source. The light radiation area (A) and the average light intensity (E) of the

reaction system are 1 × 4 cm^2 and 9.5 mW cm^{-2}, respectively. The H_2 evolution rates are obtained through the measurement of a gas chromatograph. Hence, the AQY of CdS/a-NiB photocatalyst in this work can be calculated as:

$$AQY\ (\%) = (6.6482\times10^{-5}\times R_{H_2})/(9.5\times10^{-3}\times4\times420\times10^{-9}) = 53\ \%$$

S10 Turnover Number (TON) Calculations

We have calculated the turnover number (TON) of our optimal CdS/a-NiB photocatalyst via the following equation:

$$TON=\frac{2\times \text{moles of evolved hydrogen}}{\text{moles of cocatalyst}}$$

According to the results of ICP-OES, it is found that the amount of Ni element in the CdS/a-NiB is 7.55 wt%. Therefore, for 50 mg of the CdS/a-NiB used in photocatalytic H_2-evolution reaction, the true amount of Ni element can be calculated to 3.77 mg, corresponding to 63.87 μmol. In addition, the total amount of H_2 generation of CdS/a-NiB photocatalyst was 6405 μmol for 10 h. Therefore, the TON can be calculated as following: 6405 μmol × 2 / 63.87 μmol = 200.56.

S11 Computational Details

In this work, the a-NiB model was constructed using a wide-accept "melt-and-quench" approach via dynamic simulation and geometric optimization. In detail, the c-NiB structures were melted at 1350 K (higher than the melting point temperature of crystal nickel borides) and then be quenched from 1350 K to 298 K in 300 fs,

respectively. After quenching, the obtained disordered a-NiB structures went through the geometric optimization process to gain the a-NiB model. All calculations in our work were performed within DFT using projector-augmented wave (PAW) method, as implemented in Vienna Ab initio Simulation Package (VASP) code. We have adopted conventional generalized gradient approximation (GGA) for exchange and correlation potential due to Perdew, Burke and Ernzerhof (PBE). Plane waves with a kinetic energy up to 400 eV were used to expand the wave functions. The convergence threshold was set as 10^{-5} eV for energy and 0.02 eV $Å^{-1}$ for force. A vacuum region of 20 Å was added to avoid the interactions between periodic slabs of atomic layers. A 3×3×1 Monkhorst-Pack k-point grid for Brillouin zone sampling was used during the surface calculations. The Gibbs free energy of H atom adsorption (ΔG_{H*}) was defined as $\Delta G_{H*} = \Delta E_{H*} + \Delta E_{ZPE} - T\Delta S_H$. The entropy of hydrogen at 298 K is 130 J K^{-1} mol^{-1}. Therefore, $T\Delta S_H$ was calculated to be -0.20 eV. The crystal orbital Hamilton population (-COHP) curves were calculated by using the LOBSTER package. These orbital-pair interactions can provide a quantitative measure of bond strengths. The positive and negative energy regions in the -COHP curves correspond to bonding and antibonding states, respectively. To verify the feasibility of the hydrogen transfer, the related reaction barriers of the hydrogen generation process on the a-NiB cocatalyst are deeply calculated via the climbing image nudged elastic band method (CINEB).

Table S1. The photocatalytic H_2-evolution performance and apparent quantum yields (AQY) for various cocatalyst-loaded CdS photocatalysts.

Photocatalyst	Light source	Scavenger	H_2-evolution rate (mmol $h^{-1}g^{-1}$)	AQY	Ref.
**CdS/a-NiB**	**LED (3 W)**	**lactic acid**	**12.8**	**53%**	**This work**
CdS/Pt	Xe lamp (300 W)	lactic acid	16.8	14%	[1]
Pt-CdS_x-T	Xe lamp (300 W)	lactic acid	0.8	43%	[2]
CdS/Ru	LED (3 W)	Na_2S/Na_2SO_3	1.6	9%	[3]
CdS/Ni_2P	Xe lamp (300 W)	methanol	8.2	15%	[4]
CdS/NiS-Ni_9S_8	Xe lamp (300 W)	Na_2S/Na_2SO_3	0.7	21%	[5]
CdS/MoO_2/MoS_2	Xe lamp (300 W)	lactic acid	1.2	11 %	[6]
CdS/Au@ReS_x	LED (3 W)	lactic acid	8.6	36%	[7]
CdS/PtP_2@C	LED (3 W)	lactic acid	9.7	41%	[8]
CdS/Mo_2CT_x-Au	LED (3 W)	lactic acid	2.8	22%	[9]
CdS@Au/$Ti_3C_2T_y$	Xe lamp (300 W)	lactic acid	5.4	17%	[10]

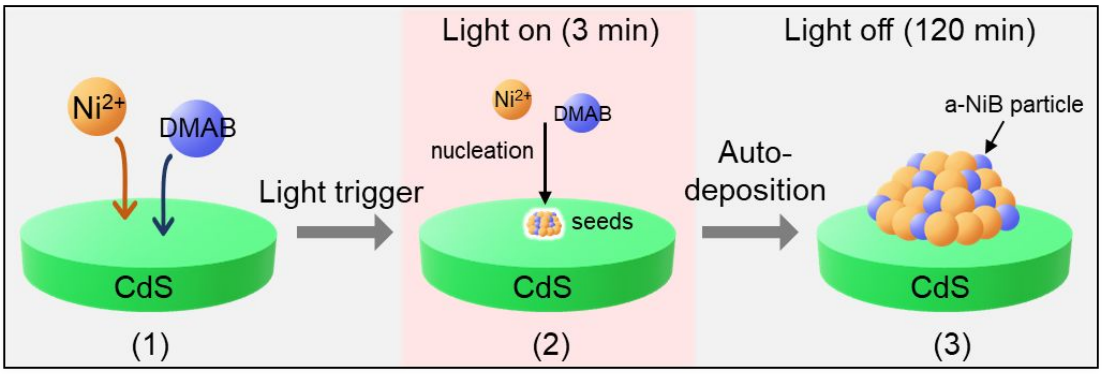

Figure S1. Schematic diagram illustrating the photo-triggered auto-deposition of a-NiB cocatalyst on CdS surface: (1) the addition of Ni source and B source, (2) the formation of a-NiB seeds via light irridiation for 3 min, and (3) the autogrowth of a-NiB seeds into a-NiB particle on CdS surface.

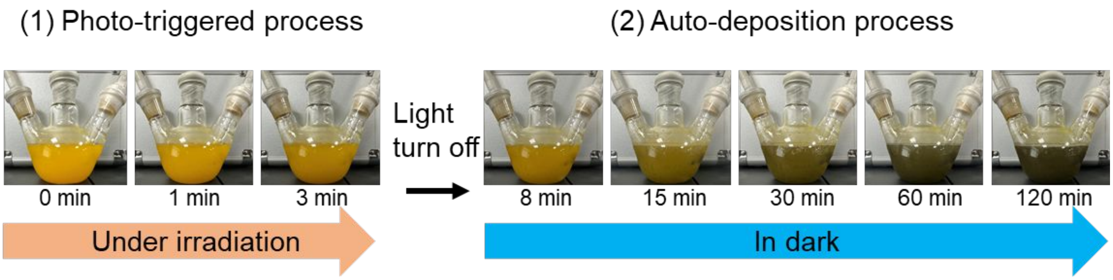

Figure S2. The color change of the CdS/a-NiB in different synthetic stages: (1) photo-triggered process and (2) auto-deposition process.

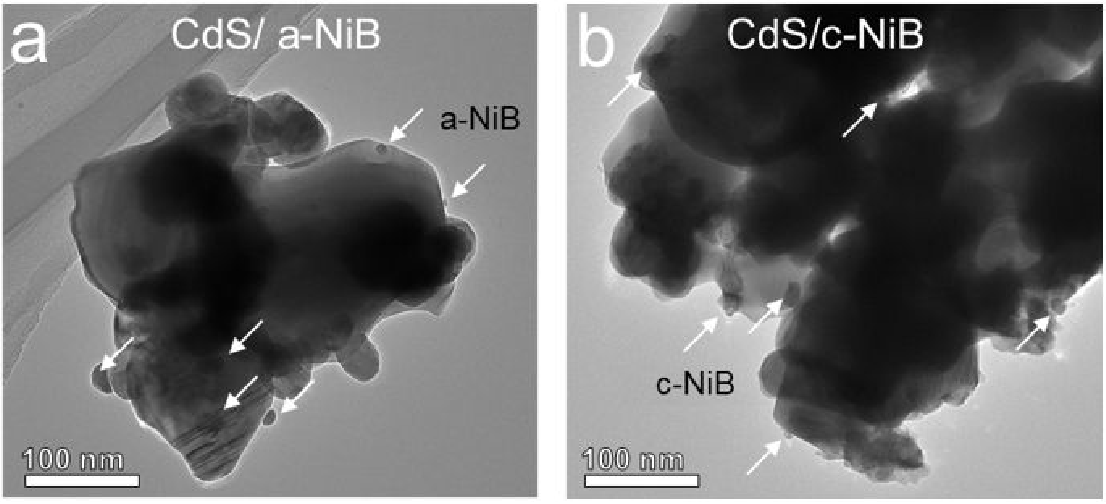

Figure S3. TEM images of (a) CdS/a-NiB, and (b) CdS/c-NiB.

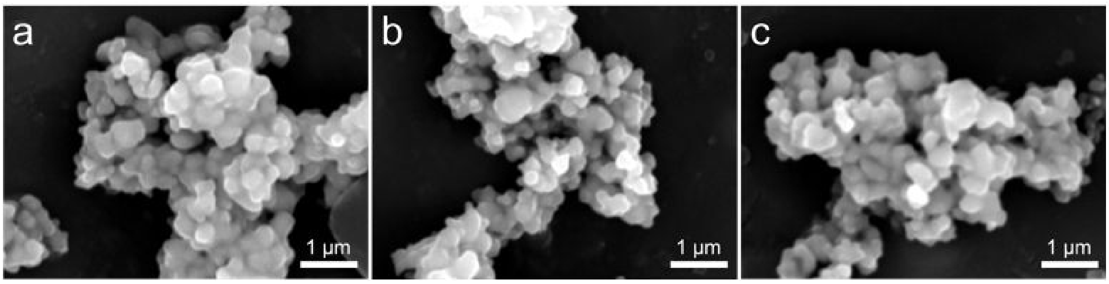

Figure S4. SEM images of (a) CdS, (b) CdS/c-NiB and (c) CdS/a-NiB.

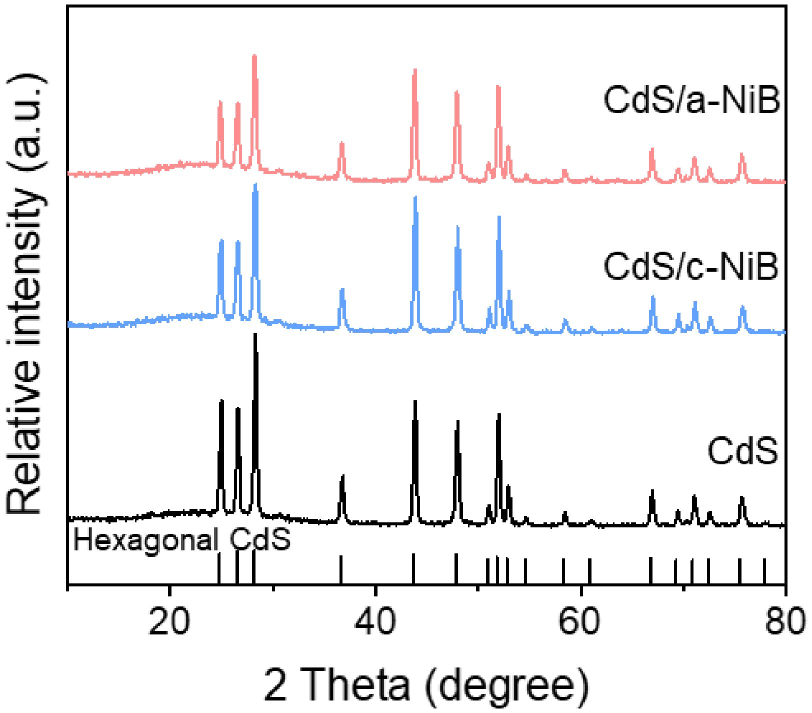

Figure S5. XRD spectra of CdS, CdS/c-NiB and CdS/a-NiB photocatalysts.

.

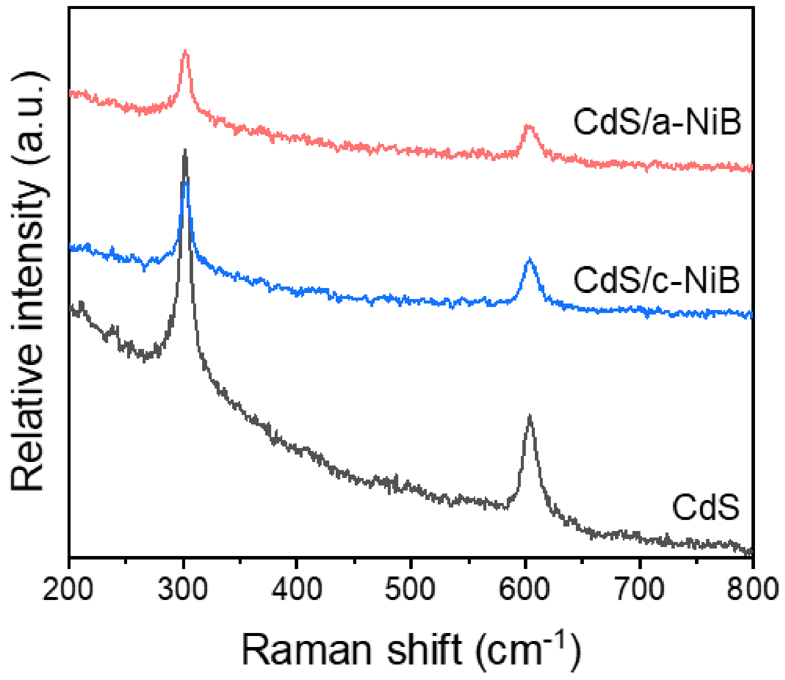

Figure S6. Raman spectra of CdS, CdS/c-NiB and CdS/a-NiB photocatalysts.

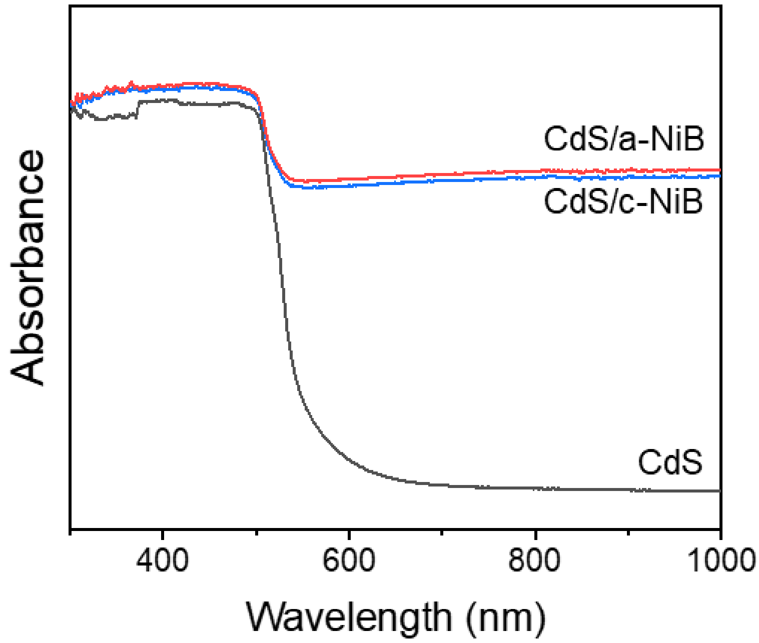

Figure S7. UV-vis spectra of CdS, CdS/c-NiB and CdS/a-NiB photocatalysts.

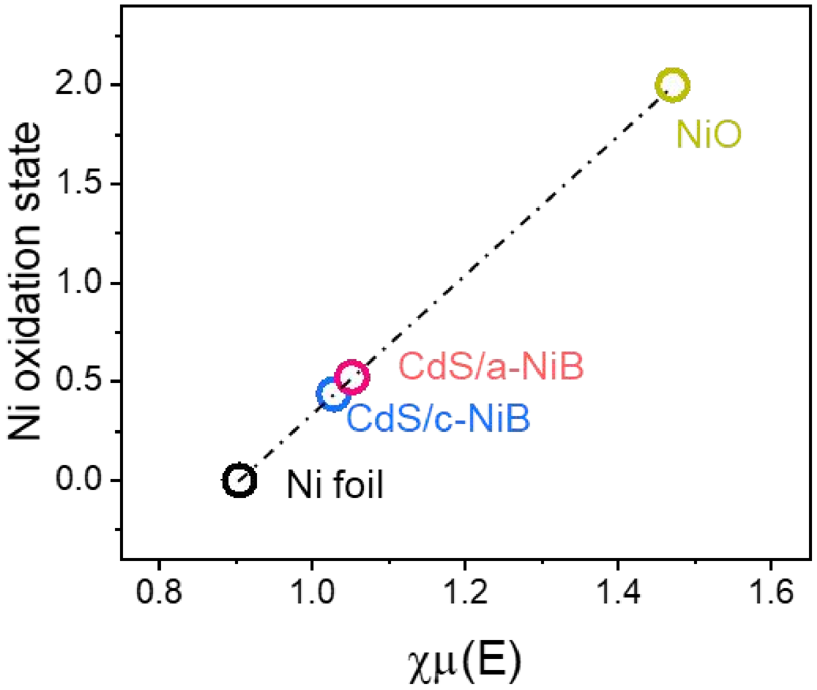

Figure S8. Ni oxidation state in CdS/c-NiB and CdS/a-NiB photocatalysts.

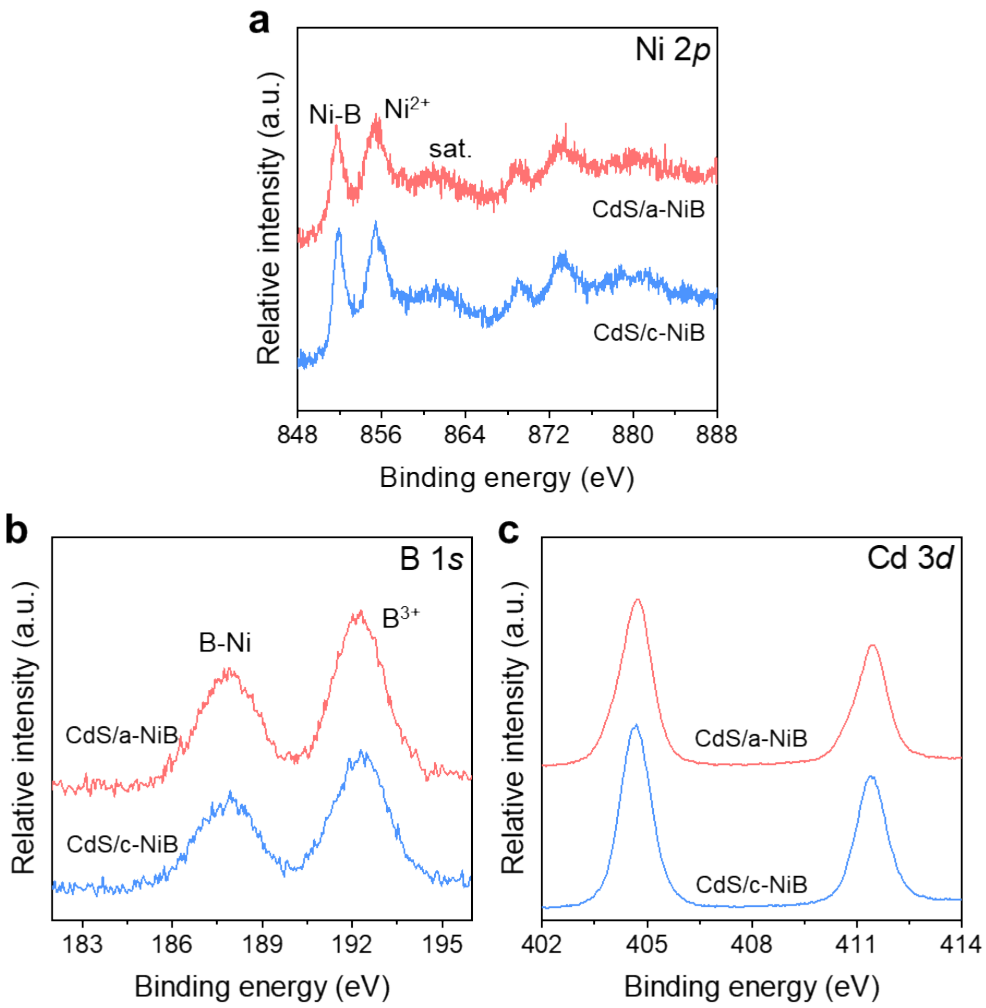

Figure S9. High-resolution XPS results of (a) Ni 2*p*, (b) B 1*s*, and (c) Cd 3*d* for CdS/c-NiB and CdS/a-NiB photocatalysts.

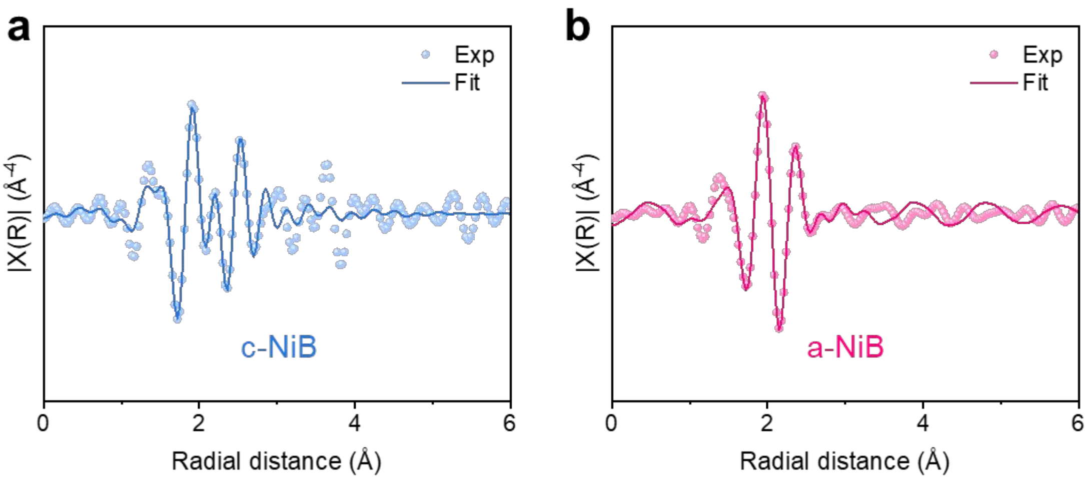

Figure S10. Fitted FT-EXAFS curves of (a) CdS/c-NiB, and (b) CdS/a-NiB.

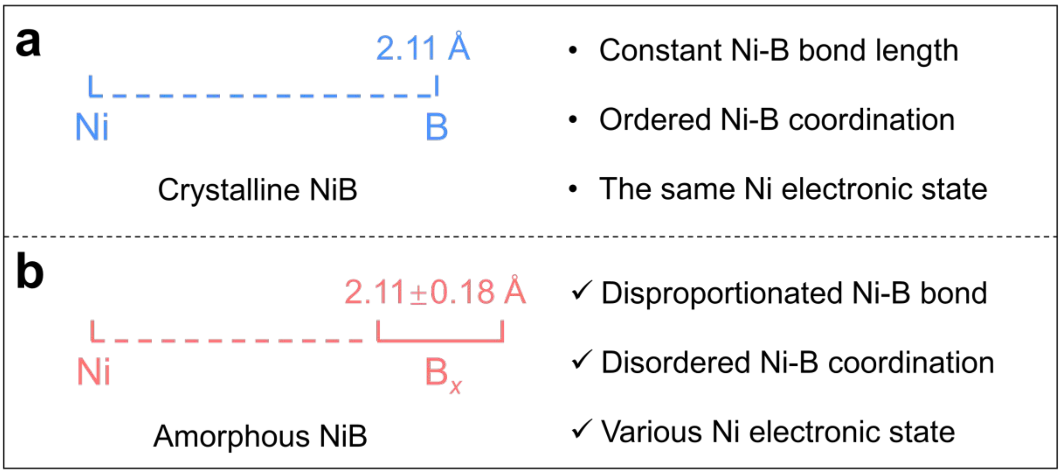

Figure S11. Properties of the Ni-B bonds in (a) crystalline NiB, and (b) amorphous NiB.

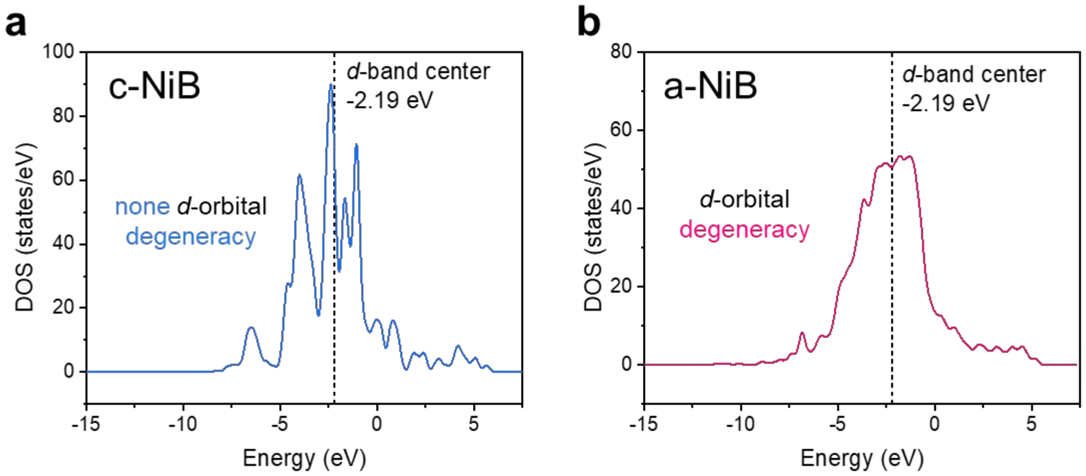

Figure S12. Ni 3*d* DOS results of (a) c-NiB, and (b) a-NiB.

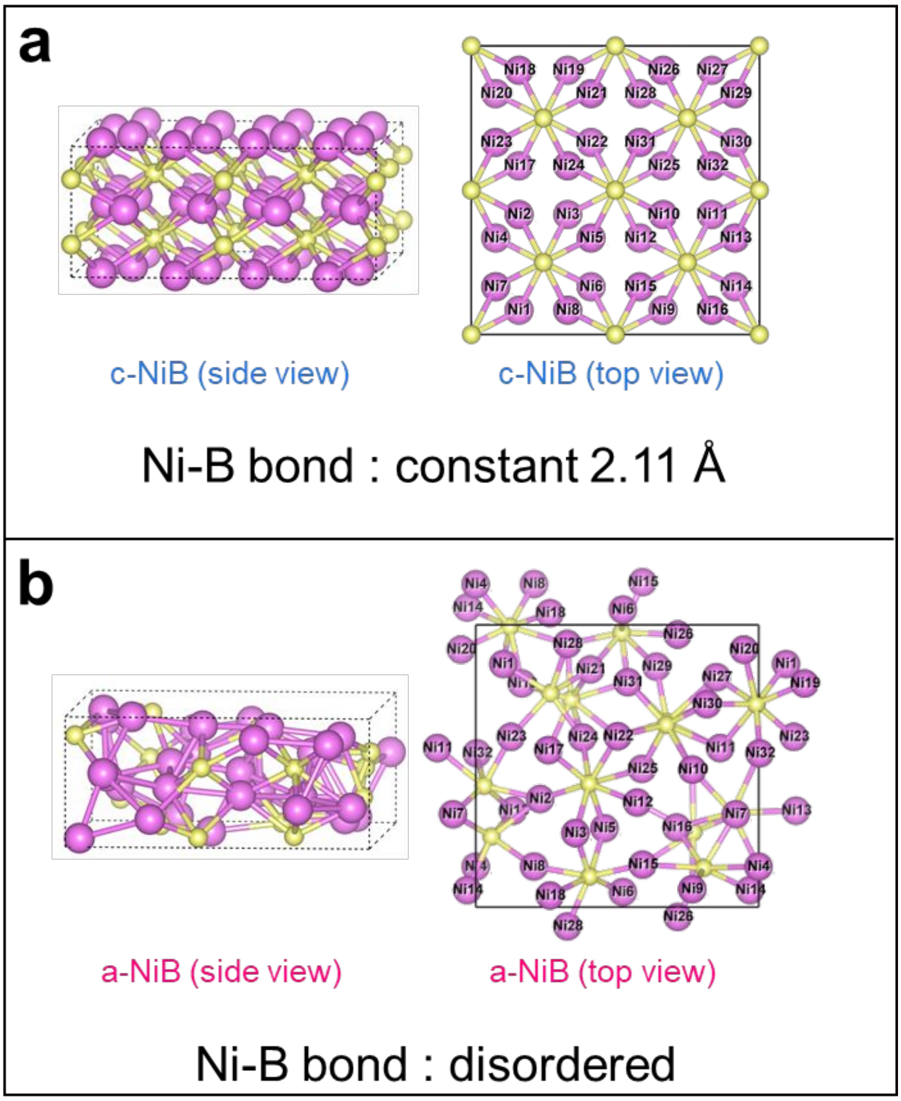

Figure S13. Models and bond distribution situation in (a) c-NiB, and (b) a-NiB.

	Ni-B length(1)	Ni-B length(2)	Ni-B length(3)	Ni-B length(4)	Ni-B length(5)	averanged Ni-B
Ni1	2.053	2.018	1.952			2.008
Ni2	2.173	2.053	2.142	2.145		2.128
Ni3	1.963	1.999	2.059			2.007
Ni4	2.034	2.258	2.04	2.244		2.144
Ni5	2.027	2.012	2.022			2.02
Ni6	2.029	2.279	2.228	2.048		2.146
Ni7	2.093	2.182	2.02	2.129	2.003	2.085
Ni8	1.944	2.019	2.03			1.998
Ni9	2.372	2.166				2.269
Ni10	2.267	2.181	2.224	2.178		2.213
Ni11	2.245	2.225	2.3	2.235	2.132	2.227
Ni12	2.027	2.022	2.211			2.087
Ni13	2.101	2.393	2.143			2.212
Ni14	2.004	2.097	2.122			2.074
Ni15	1.901	1.975	2.17	2.219		2.066
Ni16	2.374	2.05	2.265	2.01		2.174
Ni17	2.1	2.082	2.054			2.079
Ni18	1.937	2.272				2.104
Ni19	2.074	2.106	2.113	1.968	2.099	2.072
Ni20	2.201	2.19	1.964			2.118
Ni21	1.947	2.069	1.956			1.991
Ni22	2.142	2.133	2.226	2.107	2.197	2.161
Ni23	1.995	2.017	2.088	2.08		2.045
Ni24	1.965	1.925	1.916	2.034		1.96
Ni25	2.246	2.212	2.033	1.994		2.121
Ni26	2.09	1.943				2.017
Ni27	2.432	2.015	1.979			2.142
Ni28	2.243	1.945	2.144	2.038	2.082	2.09
Ni29	2.095	2.034				2.065
Ni30	2.047	2.02	1.922	1.98		1.992
Ni31	2.16	1.997	2.424	2.024		2.151
Ni32	2.465	2.023	1.983	2.073		2.136

Figure S14. Statistical Ni-B bond length data of each Ni atoms (Ni1 to Ni32) in a-NiB.

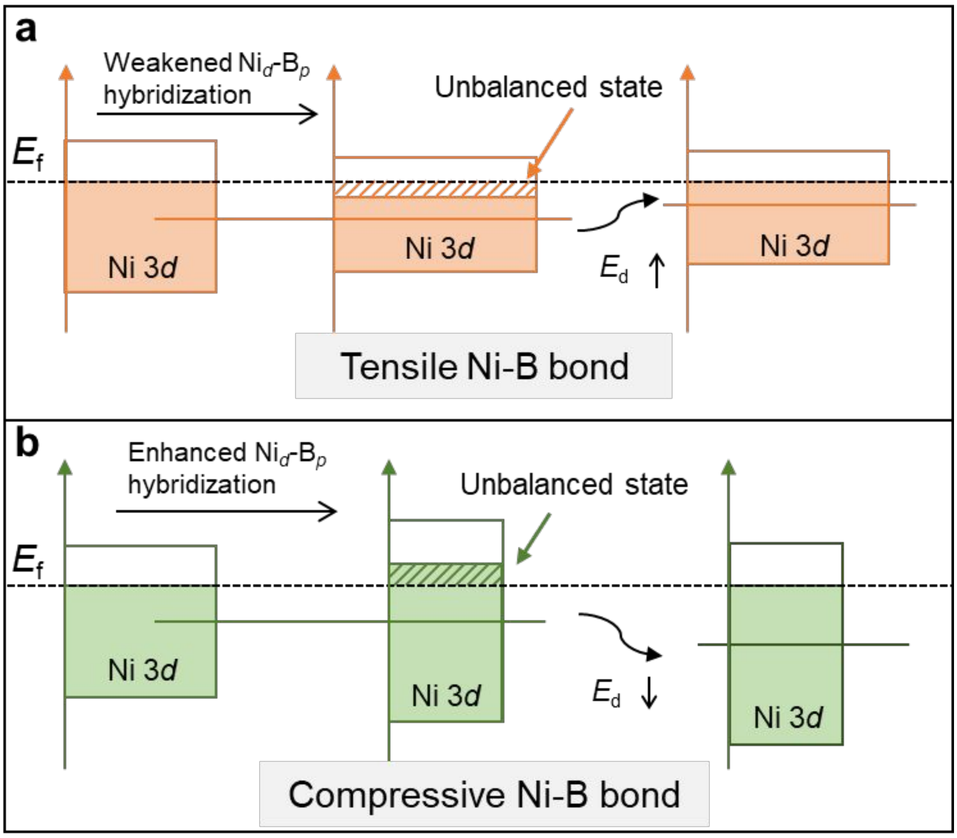

Figure S15. Schematic diagram illustrating (a) the upshift of the Ni *d*-band center via tensile Ni-B bonds, and (b) its downshift via compressive Ni-B bonds in a-NiB.

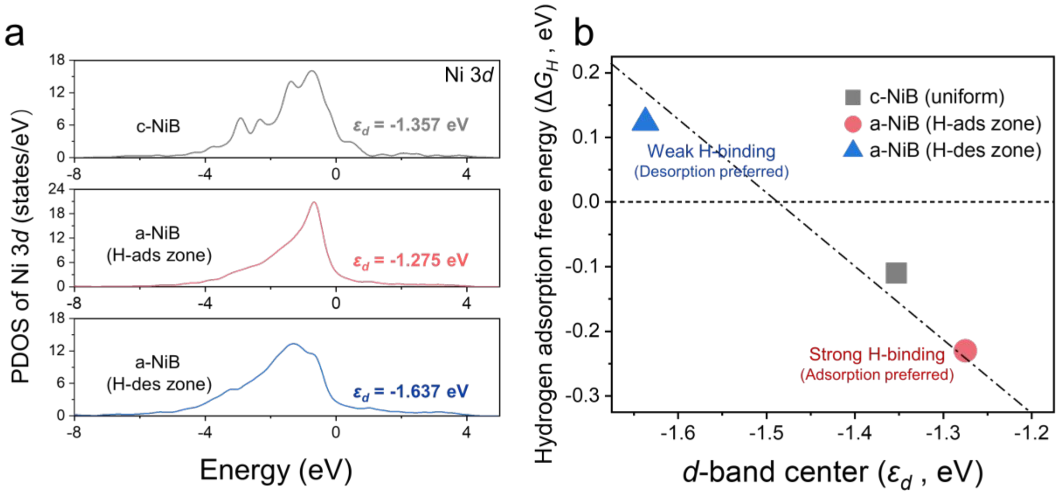

Figure S16. (a) Projected density of states (PDOS) of the Ni 3*d* orbitals for the c-NiB, the H-adsorption zone of a-NiB, and the H-desorption zone of a-NiB. The corresponding calculated *d*-band centers are indicated. (b) Linear correlation between the calculated Ni *d*-band centers and the hydrogen adsorption free energies (ΔG_H) across the different local microenvironments.

On the crystalline c-NiB surface, the active sites exhibit a uniform *d*-band center (ε_d) of -1.357 eV, corresponding to a constant hydrogen adsorption free energy of -0.120 eV. After amorphization, the a-NiB undergoes a profound electronic self-disproportionation effect, generating irregularly distributed H-ads and H-des micro-regions. In the H-ads zone of the a-NiB surface, the *d*-band center shifts up to -1.275 eV, leading to a correspondingly decreased ΔG_H of -0.236 eV. Conversely, in the H-des zone, the *d*-band center shifts down to -1.637 eV, which increases the corresponding ΔG_H to +0.123 eV.

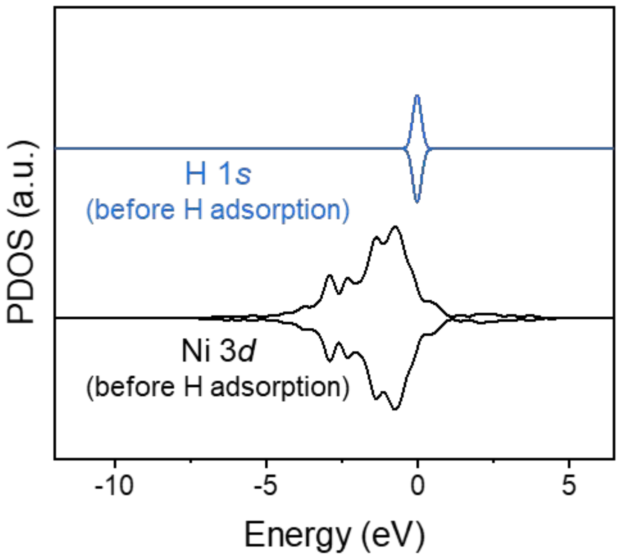

Figure S17. H 1*s* and Ni 3*d* orbital before H adsorption on Ni site in c-NiB.

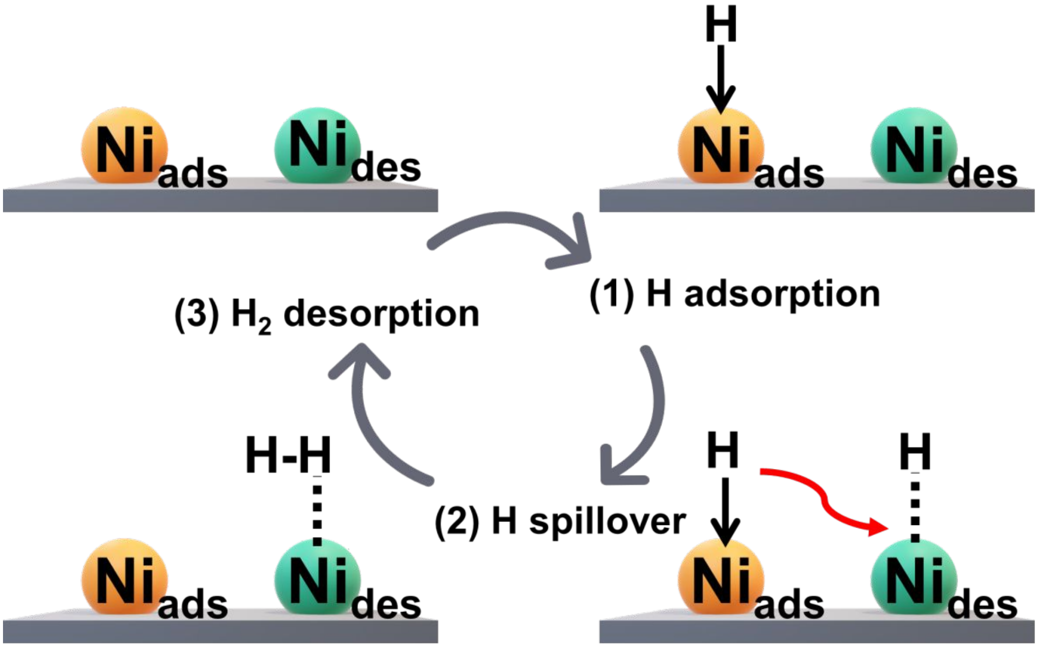

Figure S18. Graphical illustration for the possible H_2-evolution process across H_{ads} zone to H_{des} zone in a-NiB.

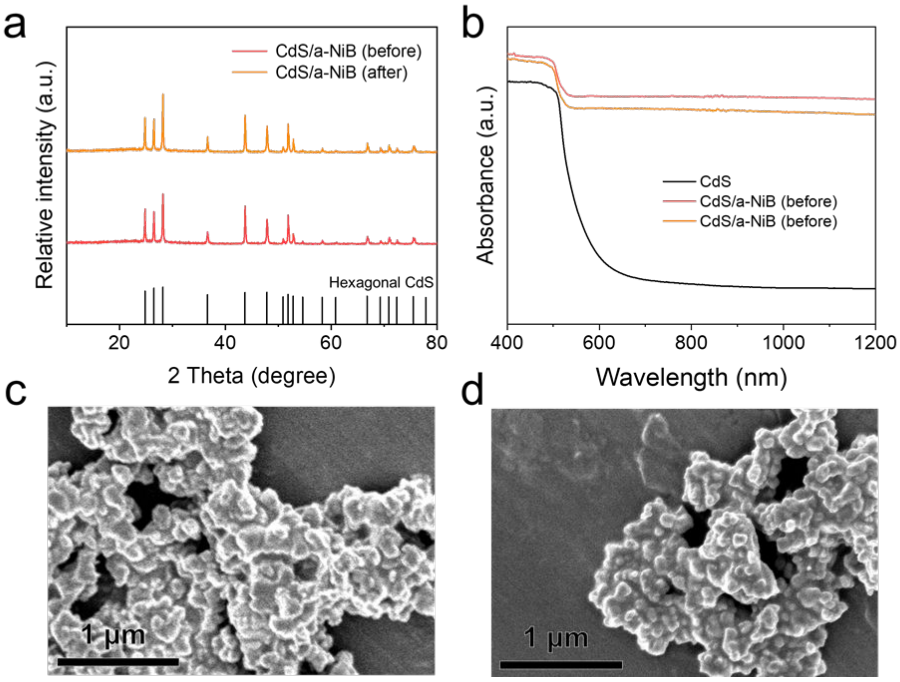

Figure S19. Post-reaction characterizations. (a) XRD patterns and (b) UV-vis spectra of the CdS/a-NiB photocatalyst before and after the long-term cycling test. SEM images of the CdS/a-NiB (c) before and (d) after the photocatalytic reaction.

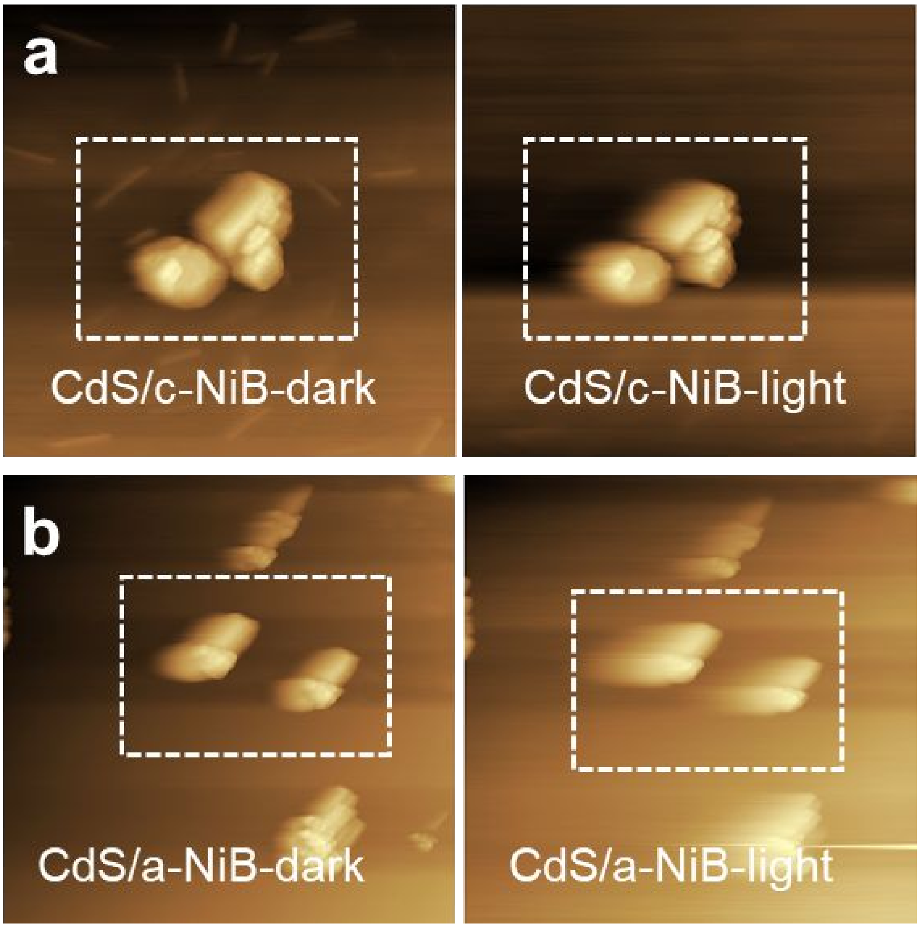

Figure S20. AFM topography images of (a) CdS/c-NiB, and (b) CdS/a-NiB in the dark (left) and light (right) condition.

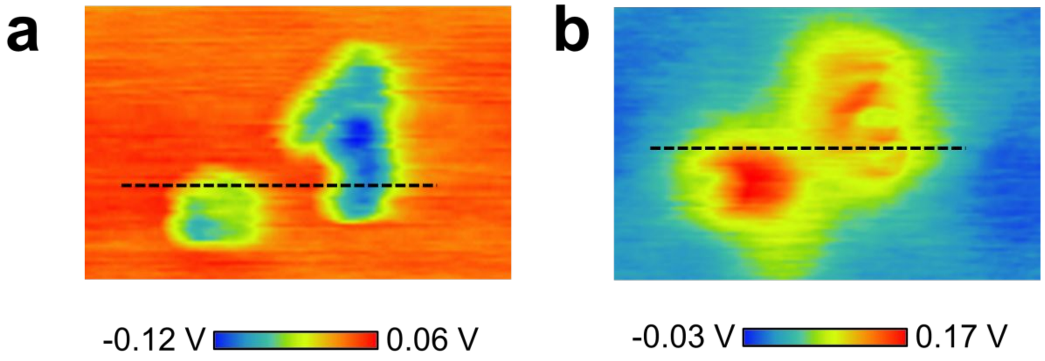

Figure S21. KPFM images of CdS/c-NiB under the (a) dark and (b) light conditions.

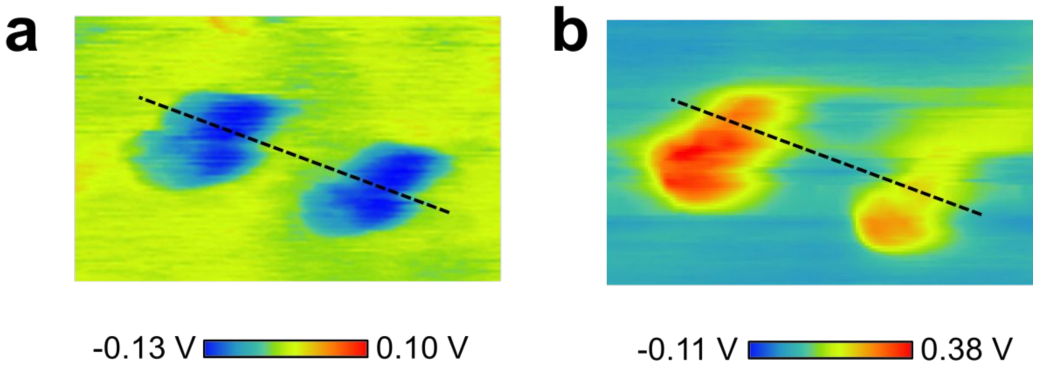

Figure S22. KPFM images of CdS/a-NiB under the (a) dark and (b) light conditions.

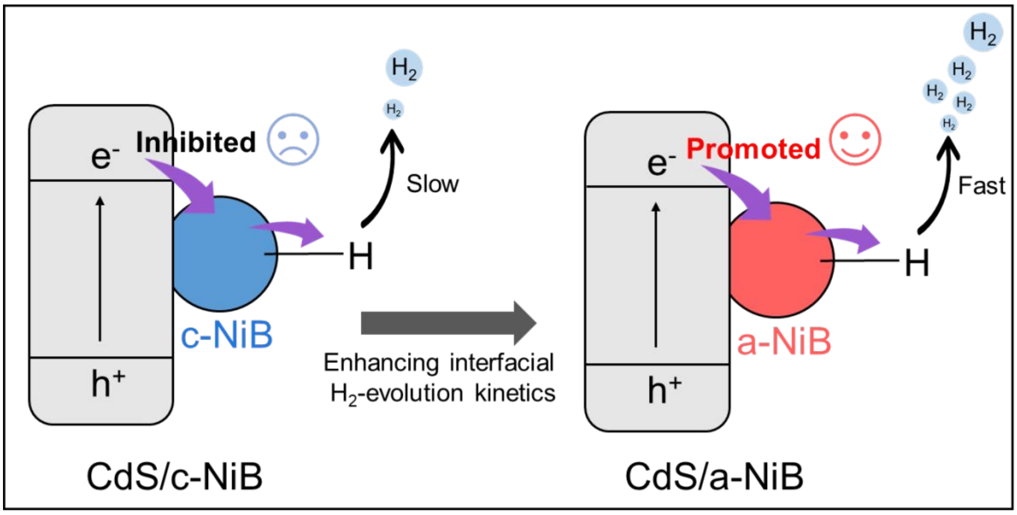

Figure S23. Schematic diagram illustrating the promoted charge carrier transfer from CdS to a-NiB cocatalyst via enhancing interfacial H_2-evolution kinetics.

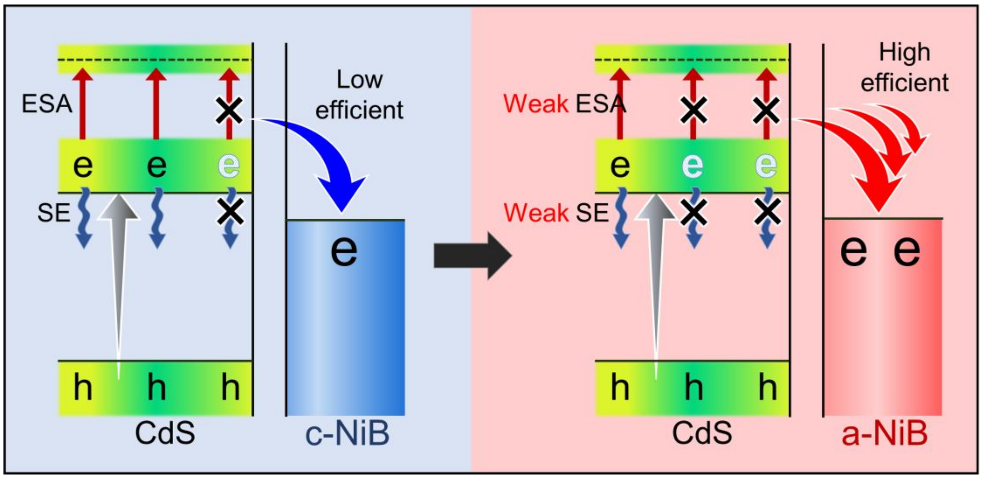

Figure S24. Diagram illustration for the high-efficient charge carrier transfer from CdS to a-NiB cocatalyst to weaken the ESA and SE process in the conductive band.

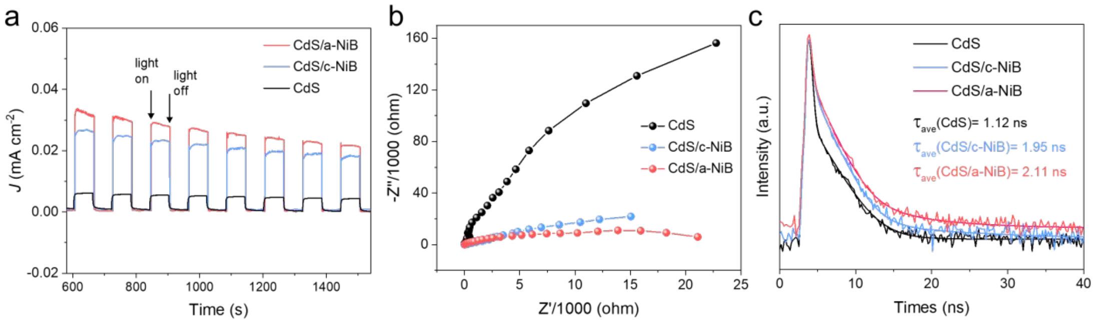

Figure S25. (a) Transient photocurrent responses, (b) electrochemical impedance spectroscopy, and (c) transient photoluminescence results of various samples.

References

(1) Zhang, J.; Cai, P.; Lin, J. Modulation of the Band Bending of CdS by Fluorination to Facilitate Photoinduced Electron Transfer for Efficient H_2 Evolution over Pt/CdS. *J. Phys. Chem. C* **2022**, *126*, 7896–7902.

(2) Tang, Y.; Jia, X.; Guo, Y.; Geng, Z.; Wang, C.; Liu, L.; Zhang, J.; Guo, W.; Tan, X.; Yu, T.; Ye, J. Surface Unsaturated Sulfur Modulates Pt Sub-Nanoparticles on Tandem Homojunction CdS for Efficient Electron Extraction. *Adv. Energy Mat.* **2023**, *14*, 2203827.

(3) Stavitskaya, A. V.; Kozlova, E. A.; Kurenkova, A. Y.; Glotov, A. P.; Selischev, D. S.; Ivanov, E. V.; Kozlov, D. V.; Vinokurov, V. A.; Fakhrullin, R. F.; Lvov, Y. M. Ru/CdS Quantum Dots Templated on Clay Nanotubes as Visible-Light-Active Photocatalysts: Optimization of S/Cd Ratio and Ru Content. *Chem. Eur. J.* **2020**, *26*, 13085.

(4) Chen, A.; Yang, X.; Shen, L.; Zheng, Y.; Yang, M. Directional Charge Pumping from Photoactive P-doped CdS to Catalytic Active Ni_2P via Funneled Bandgap and Bridged Interface for Greatly Enhanced Photocatalytic H_2 Evolution. *Small* **2024**, *20*, 2309805.

(5) Xie, Y.; Chang, J. Zheng, P.; Zhang, L.; Xie, T.; Jiang, R.; Zhang, Z.; Yang, Y.; Zou, M.; Yin, L.; Zhen, C.; Han, F.; Ba, K.; Xu, G. Evidence for an Interface of Hybrid Cocatalysts Favoring Photocatalytic Hydrogen Evolution Kinetics. *ACS Appl. Mater. Interfaces* **2023**, *15*, 59309.

(6) Bie, C.; Zhu, B.; Wang, L.; Yu, H.; Jiang, C.; Chen, T.; Yu, J. A Bifunctional CdS/MoO_2/MoS_2 Catalyst Enhances Photocatalytic H_2 Evolution and Pyruvic Acid Synthesis. *Angew. Chem. Int. Ed.* **2022**, *61*, e202212045.

(7) Yin, X.; Gao, D.; Zhang, J.; García, H.; Yu, J.; Yu, H.; Plasmon-Induced Ultrafast Interfacial

Charge Transfer for Enhanced Photocatalytic Hydrogen Evolution. *J. Am. Chem. Soc.* **2025**, *147*, 34881–34890.

(8) Xu, J.; Zhong, W.; Gao, D.; Wang, X.; Wang, P. Phosphorus-Enriched Platinum Diphosphide Nanodots as A Highly Efficient Cocatalyst for Photocatalytic H_2 evolution of CdS. *Chem. Eng. J.* **2022**, *439*, 135758.

(9) Ke, X.; Wang, P.; Wang, X.; Chen, F.; Yu, H. Releasing Au Electrons to Mo Site for Weakened Mo-H Bond of Mo_2C MXene Cocatalyst Toward Improved Photocatalytic H2 Production. *Small* **2024**, *20*, 2405378.

(10) Wei, P.; Chen, Y.; Zhou, T.; Wang, Z.; Zhang, Y.; Wang, H.; Yu, H. Jia, J.; Zhang, K.; Peng, C. Manipulation of Charge-Transfer Kinetics via $Ti_3C_2T_x$ (T = -O) Quantum Dot and N-Doped Carbon Dot Coloading on CdS for Photocatalytic Hydrogen Production. *ACS Catal.* **2023**, *13*, 587.
